# Panacis Quinquefolii Radix: A Review of the Botany, Phytochemistry, Quality Control, Pharmacology, Toxicology and Industrial Applications Research Progress

**DOI:** 10.3389/fphar.2020.602092

**Published:** 2020-12-08

**Authors:** Liu Yang, Ajiao Hou, Jiaxu Zhang, Song Wang, Wenjing Man, Huan Yu, Senwang Zheng, Xuejiao Wang, Songtao Liu, Hai Jiang

**Affiliations:** Key Laboratory of Chinese Materia Medica, Ministry of Education, Heilongjiang University of Chinese Medicine, Harbin, China

**Keywords:** Panacis Quinquefolii Radix, phytochemistry, quality control, pharmacology, toxicology, industrial applications

## Abstract

On January 2, 2020, The National Health Commission and the State Administration for Market Regulation listed Panacis Quinquefolii Radix (PQR) as a medicinal and food homologous product. PQR is the dry root of *Panax quinquefolium* L., which has the functions of replenishing qi and nourishing Yin, clearing heat and producing body fluid. It is often used for qi deficiency and Yin deficiency, heat exhaustion, asthma and phlegm, dry mouth and pharynx. PQR is sweet, slightly bitter and cool in nature, and enter the heart, lung and kidney meridian exerts the remedial and hygienical effect. At present, active components such as saponins, flavonoids, fatty acids, polyalkynes, volatile oils and other nutrients such as amino acids, carbohydrates, vitamins and trace elements have been isolated from PQR. Modern pharmacological studies have shown that PQR has the effects of hypoglycemic, antihypertensive, anti fatigue, anti-oxidation, anti-tumor, immunomodulatory, neuroprotective and so on. In addition, PQR is recognized as a health care product to strengthen the body and dispel diseases. It is not only the raw material of Traditional Chinese medicine preparations, but also the treasure of dietary therapy and herbal cuisine. This study not only reviewed the botany, phytochemistry and pharmacology of PQR, but also summarized its quality control, toxicity and industrial applications for the first time. This paper not only summarizes the development status of PQR, but also analyzes the shortcomings of the current research on PQR, and puts forward the corresponding solutions, in order to provide reference for future scholars to study PQR.

## Introduction

**FIGURE 1:**
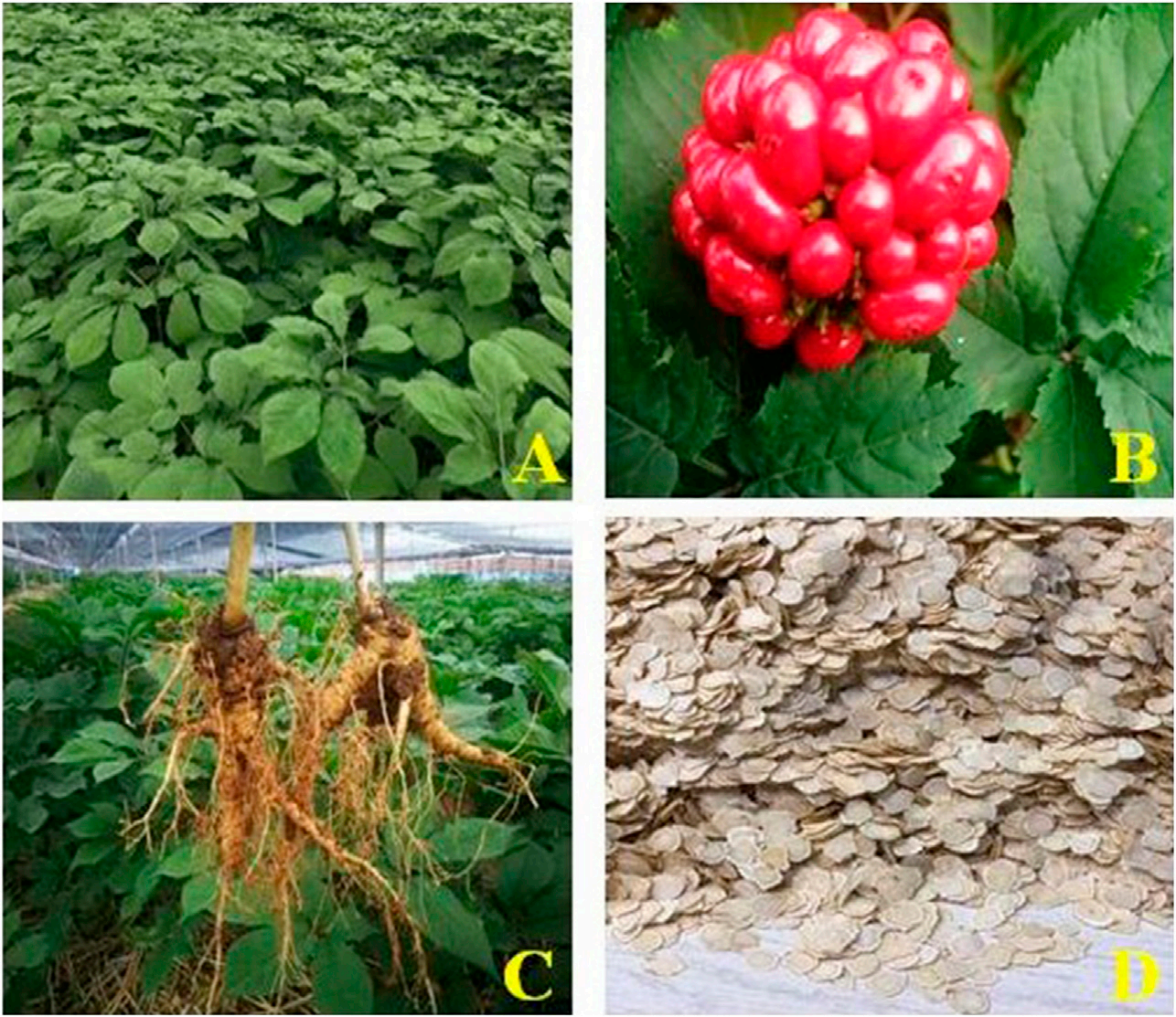
*Panax quinquefolium* L. stem-leaves **(A)**, *Panax quinquefolium* L. fruit **(B)**, *Panax quinquefolium* L. roots **(C)** and *Panax quinquefolium* L. slices **(D)**.

Panacis Quinquefolii Radix (PQR), has a Chinese name of “Xiyangshen,” (Figure 1), derives from the dry root of *Panax quinquefolium* L., which is sweet, slightly bitter and cool in nature and has the functions of replenishing qi and nourishing Yin, clearing heat and producing body fluid. PQR was listed as a medicinal and food homologous product by the National Health Commission and the State Administration for Market Regulation on January 2, 2020. PQR is native to Montreal, Quebec and Vancouver mountains in Canada, and Wisconsin, Missouri and New York in the eastern United States and West Virginia, Oregon and California, etc. (Qu, 2009).

PQR was first published in “ New Compilation of Materia Medica” (本草从新), which can prove that PQR has been used in China for more than 200 years (Chen, 1995). For a long time, China has been dependent on the import of PQR, and most of the medicines are limited to nourishing and strengthening or used as a placebo for patients with advanced cancer, which limits the medicinal range of PQR and makes this precious medicinal material fail to play its full role. PQR was introduced and planted in China in 1970s, now it is widely cultivated in Jilin, Heilongjiang, Shaanxi, Henan, Jiangxi, Liaoning, Beijing, Fujian and other places (Jin and Fan, 1992; Ma and Rui, 2019). At present, China has developed into the third largest PQR producing country in the world after the United States and Canada, and the world’s largest PQR consumer country.

In recent years, scholars at home and abroad have studied PQR. Chemical studies have shown that PQR contains active components such as saponins (Wang M. Y. et al., 2020), polysaccharides (Zhai et al., 2015), flavonoids (Zhen, 2012), fatty acids, polyalkynes (Fujimoto et al., 1991), sterols, volatile oils (Liu X. F. et al., 2016) and nutrients such as amino acids, carbohydrates, vitamins (Wang Y. et al., 2013), trace elements (Zhao et al., 2012). Modern pharmacological studies have shown that PQR has the effects of hypoglycemia (Jenkins et al., 2017), hypotension (Stavro et al., 2006), antioxidant (Liu et al., 2019), anti-tumor (Chang et al., 2018), immune regulation (Jiang et al., 2020), neuroprotection (Shin et al., 2016) and so on. It is commonly used in the treatment of cancer (Chen et al., 2016), hypertension (Jovanovski et al., 2020), diabetes (Vuksan et al., 2019), myocardial ischemia (Yu et al., 2020), gastrointestinal tract and other diseases (Liu et al., 2020). Furthermore, PQR is also widely used in health care medicines, health food, health care cosmetics and other fields, such as drinks, biscuits, medicated meals, facial mask, etc. (Li et al., 2015; Yim et al., 2015), they have the functions of enhancing immunity, relieving physical fatigue (Qi et al., 2014), delaying aging (Song et al., 2019),anti-radiation (Luo et al., 2014) auxiliary protective function for chemical liver injury (Jiang et al., 2020)

PQR is a valuable medicinal material, which has been favored by people for its unique medical and health care functions. In this study, 1,742 literatures about PQR from 1982 to now were collected and consulted, in addition to repetitive and irrelevant literatures, 172 articles were reviewed in this study on botany, phytochemistry, quality control, pharmacology, toxicology and industrial applications of PQR. In this study, the ingredients in PQR were displayed in detail, and their biological activities were summarized. For the first time, the research on quality control, toxicity and industrial applications were reviewed. Not only the development status of PQR was briefly reviewed, but also the shortcomings of the current research were discussed and the corresponding solutions were proposed, so as to provide important information for future scholars to study, development and utilization of PQR. In a word, this review has important reference value for the further study of PQR.

## Botany

According to the Chinese Pharmacopoeia (2020 edition), PQR is spindle shaped, cylindrical or conical, with a length of 3–12 cm and a diameter of 0.8–2 cm. Its surface is light yellow-brown or yellow-white, with transverse annular lines and linear lenticel like protuberances, and have fine light longitudinal wrinkles and fibrous root marks. There are one or several lateral roots in the middle and lower part of the main root of PQR, most of which have been broken. Some PQR have rhizomes at the upper end, and the links are obvious. The stem marks are round or semicircular, and most of them have been broken.PQR is heavy in quality, solid in texture and not easy to be broken. The cross section of PQR is flat, pale yellow and white, with slightly powdery appearance. There are yellow-brown dot resin channels in the skin, cambium ring with brownish yellow color, and wood part with slightly radial texture. PQR has a small and unique smell and a slightly bitter and sweet taste. As the species of PQR are precious and wild resources are scarce, the majority of PQR in the market are cultivated products, and the difference between its traits and wild products is shown in Table 1.

**TABLE 1:** The difference of characters of wild and cultivated PQR.

Characters	Wild PQR	Cultivated PQR
Shape	Similar size to silkwormmoth	As thick as a thumb, cylindrical or long spindleshaped, about 3–12 cm long, 0.8–2 cm in diam
Any branch or not	No	Occasionally exist (3–12 cm long, 0.8–2 cm in diam)
Color	Earth yellow and smooth scarfskin, white light inside	Light brown or yellowish white surface, white light inside
Body	Dense ringlike fine stripes, especially at the top	Fine stripes less than wild one, visible scarce vertical lines at the lower part occasionally
Texture	Light and hard	More solid
Odor	Taste slightly sweet and bitter	Relatively weaker than wild American ginseng

PQR, panacis quinquefolii radix.

“Chinese Materia Medica” (中华本草) records: PQR likes humid environment, should avoid strong light irradiation. Therefore, the cultivation of PQR needs to build a shading shed, the light transmittance of the shed is 20–25%. The light transmittance should be higher in spring (about 22%) and lower in summer, which can reduce the disease. And the soil for PQR cultivation should be sandy loam with good water permeability and fertility, mixed with large coarse sand, rather than clay or low-lying areas with poor drainage and easy to accumulate water.

## Nutritional and Physiochemical Composition

### Nutriment

At present, the world medicine is developing toward the combination of curative effect and nutrition. Modern research has confirmed that PQR contains protein, carbohydrate, vitamin, fat and other essential nutrients and macro elements, trace elements. Undoubtedly, they are valuable resources for nourishment, fitness, nutrition and medicine, and should be fully utilized.

### Amino Acid

PQR is rich in nutrients and contains a variety of amino acids, (As shown in Table 2), which have a positive effect on the human body. Zhang et al. determined that there were 18 kinds of amino acids (12.838 g/L) in PQR berries, among which 10 kinds were essential and semi-essential amino acids for human body (Cao, 2004). Zheng et al. isolated a special amino acid from PQR, denciemne, which is a major component for promoting blood circulation (Zheng and Deng, 1989). The amino acid contents in roots, stems, leaves, buds and seeds of PQR at different ages were analyzed. The results showed that the amino acid content of PQR decreased with the increase of PQR age. The contents of total amino acids in four-year-old PQR were as follows: flower bud (13.922%) > leaf (11.723%) > root (5.932%) > seed (5.006%) > stem (3.372%) (Li et al., 1985). Therefore, the flower buds and leaves of PQR contain a lot of nutrients. However, at present, the flower bud and leaf of PQR are rarely used, and it has not been deeply applied in nutrition and health care. Most of the flower bud and leaf resources are abandoned and cannot be fully utilized. In the future, the flower buds and leaves can be developed into PQR tea with health functions, or the flower buds of PQR can be made into PQR flower-cakes and biscuits, so that the resources of PQR can be better developed and utilized.

**TABLE 2:** The component of amino acids in panacis quinquefolii radix.

No	Name	Source	References
1	Alanine	Root, stems, leaves, fruit, flower bud	Li et al. (1985)
2	Arginine	Root, stems, leaves, fruit, flower bud	Li et al. (1985)
3	Aspartic acid	Root, stems, leaves, fruit, flower bud	Li et al. (1985)
4	Cystine	Root, stems, leaves, fruit, flower bud	Li et al. (1985)
5	Dencichine	Whole PQR herb	Zheng and Deng (1989)
6	Glutamic acid	Root, stems, leaves, fruit, flower bud	Li et al. (1985)
7	Glycine	Root, stems, leaves, fruit, flower bud	Li et al. (1985)
8	Histidine	Root, stems, leaves, fruit, flower bud	Li et al. (1985)
9	Isoleucine	Root, stems, leaves, fruit, flower bud	Li et al. (1985)
10	Leucine	Root, stems, leaves, fruit, flower bud	Li et al. (1985)
11	Lysine	Root, stems, leaves, fruit, flower bud	Li et al. (1985)
12	Methionine	Root, stems, leaves, fruit, flower bud	Li et al. (1985)
13	Phenylalanine	Root, stems, leaves, fruit, flower bud	Li et al. (1985)
14	Proline	Root, stems, leaves, fruit, flower bud	Li et al. (1985)
15	Serine	Root, stems, leaves, fruit, flower bud	Li et al. (1985)
16	Threonine	Root, stems, leaves, fruit, flower bud	Liet al. (1985)
17	Tryptophan	Root, stems, leaves, fruit, flower bud	Cao (2004)
18	Tyrosine	Root, stems, leaves, fruit, flower bud	Li et al. (1985)
19	Valine	Root, stems, leaves, fruit, flower bud	Li et al. (1985)

### Polysaccharides

The proportion of polysaccharides in PQR is higher, and monosaccharides include glucose, fructose and sorbose, and the oligosaccharides include ginseng trisaccharide, maltose and sucrose (Chen and Cui, 2002). Zhang’s study showed that the average carbohydrate content of PQR from different regions was: reducing sugar 3.70%, oligosaccharide 15.40%, polysaccharide 49.74%, total sugar 68.84%. The results showed that 11.74–19.86% of water-soluble pectin could be extracted from PQR. The water-soluble pectin was composed of galacturonic acid, galactose (Gal), glucose (Glu), arabinose (Ara), xylose (Xyl), rhamnose (Rha) and a few unknown sugars (Zhang and Li, 1989). Liang et al. showed that the total sugar in PQR include starch, pectin, oligosaccharide and monosaccharide. The total amount is 68.2%–74.3% (Liang et al., 1989). In addition, some scholars also isolated a variety of polysaccharides from PQR, as shown in Table 3. However, there are few studies on the isolation and identification of polysaccharides in PQR. The possible reason is that the components of polysaccharides are complex and it is difficult to identify the polysaccharides by modern analytical methods. Polysaccharides from PQR have many pharmacological functions, such as immune regulation, anti-oxidation, anti-virus, anti-inflammatory, etc. In the future, researchers can develop more new instruments and methods, improve the identification system of polysaccharides, and develop monosaccharide drugs to facilitate clinical use. At the same time, the development of PQR polysaccharides into health food can also be used as a dietary supplement for human body, which can obtain a higher degree of acceptance than drugs.

**TABLE 3:** Composition of panacis quinquefolii radix polysaccharides.

No	Name	Monosaccharide composition	Molecular weight	References
1	AEP-1	Glc,Gal and GalA in a molar ratio of 4.67:0.97:3.92	Unknow	Yu et al. (2014)
2	AEP-2	Ara, man, Gal, Glc and GalA with a relative molar ratio of 1.03:0.76:1.68:3.02:3.65	Unknow	Yu et al. (2014)
3	GSP	Rha, Ara, Gal, Glc, Gal in a ratio of 1:4:8:8:50	8.54 × 10^4^	Wang et al. (2015a)
4	PPQ	Glc, Gal in the ratio of 2.1:1	5.4 × 10^4^	Wang et al. (2015a)
5	PPQI	Ara, Gal, Glc in the ratio of 31.5:58.5:10.0 and 38% urnoic acid	6.1 × 10^5^	Ma et al. (2000)
6	PPQII	Ara, Gal, Glc in the ratio of 34.4:59.5:5.5 and 32% uronic acid	4.7 × 10^5^	Ma et al. (2000)
7	PPQIII	Ara, Gal, Glc in the ratio of 43.6:46.2:10.0 and 35% uronic acid	3.9 × 10^5^	Ma et al. (2000)
8	PPQIV	Ara, Gal in the ratio of 29.3:70.2 and 60% uronic acid	2.8 × 10^5^	Ma et al. (2000)
9	PPQA2	Ara, Rha, Man, Gal, Glu ,galacturonic acid and glucuronic acid in a molar ratio of 8.0:4.0:2.9:7.2:12.5:26.6:38.8	2.3 × 10 ^4^	Wang et al. (2015a)
10	PPQA4	Ara, Rha, Man, Gal, Glu and glucuronic acid in a molar ratio of 19.7:5.1:8.1:23.9:41.3:2.0	1.2 × 10 ^5^	Wang et al. (2015a)
11	PPQA5	Ara, Rha, Man, Gal, Glu ,galacturonic acid and glucuronic acid in a molar ratio of 8.5:3.2:5.3:10.8:32.4:15.5:24.4	5.3 × 10 ^3^	Wang et al. (2015a)
12	PPQ-d	Ara, Gal in the ratio of 1:1.1 and 60% uronic acid	2.1 × 10^4^	Wang et al. (2015a)
13	PPQN	Glc, Gal in the ratio of 1:1.15	3.1 × 10^3^	Wang et al. (2015a)
14	Quinquefolan A	Man, Glc in the ratio of 1.0:2.3 and 10.8% uronic acid	>2.0 × 10^6^	Oshima et al. (1987)
15	Quinquefolan B	Man, Glc in the ratio of 1.0:5.5 and 11.7% uronic acid	>2.0 × 10^6^	Oshima et al. (1987)
16	Quinquefolan C	Xyl and 7.1% uronic acid	>2.0 × 10^6^	Oshima et al. (1987)
17	SPS-1	Gal, Ara, Xyl, Man, Glu, galacturonic acid in the ratio of 4.5:4.2:1.1:1.3:1.5:4.0	3.62 × 10^6^	Yu (2016)
18	SPS-2	Glu, Gal, Ara, Xyl, Man, galacturonic acid in the ratio of 7.9:7.8:4.2:1.5:1.9:5.7	9.70 × 10^6^	Yu (2016)
19	SPS-3	Ara, Rha, Xyl, Man, Gal, Glu, galacturonic acid, glucuronic acid in the ratio of 5.2:3.0:2.8:1.5:3.4:3.0:6.5:2.3	5.12 × 10^5^	Yu (2016)
20	WPS-1	Ara, Gal,Glu in the ratio of 5.2:6.6:20.8	1.54 × 10^6^	Yu (2016)
21	WPS-2	Ara, Man, Gal,Glu in the ratio of 3.7:1.2:2.4:3.8	1.41 × 10^6^	Yu (2016)

### Fatty Acid

PQR is rich in fatty acids. (As shown in Table 4). Li et al. showed that the saturated fatty acid with the highest content in the flower bud of PQR was palmitic acid, and the unsaturated fatty acid with the highest content was linoleic acid (Li et al., 1999). Yang et al. showed that the fatty acid with the highest content in the root of PQR was methyl linoleate (59.56%) (Yang et al., 2008). Linoleic acid is an essential fatty acid for human body. Linoleic acid is very important for cholesterol metabolism. Only when cholesterol is combined with linoleic acid, can it run in the body and metabolize normally. If linoleic acid is deficient, cholesterol will combine with saturated fatty acid and deposit in human body. Moreover, linoleic acid can protect skin from radiation damage. In addition, linoleic acid also has a strong effect on reducing the concentration of LDL cholesterol. In Chinese Pharmacopoeia, ethyl linoleic acid pills and drops are still used to prevent and treat hypertension, atherosclerosis and coronary heart disease. However, most of the articles on anti-radiation, anti-hypertension and anti-cardiovascular diseases of PQR mainly focus on the study of the crude PQR and ginseng saponin, and few studies have conducted in-depth discussion on the composition, pharmacological effect and mechanism of fatty acid compounds in PQR. In the future, scholars can try to extract linoleic acid from PQR, and then apply it to the treatment of some diseases. Future scholars should not only study the saponins, they should pay more attention to other active components and analyze the overall characteristics of PQR.

**TABLE 4:** The fatty acids compounds of panacis quinquefolii radix.

No	Name	Source	References
1	Butyl octyl 1,2-phenylenedicarboxylate	Root	Yang et al. (2008)
2	1-Cyclopropyl-ketene	Root	Yang et al. (2008)
3	Decanoic acid, 8-methyl-metlhyl	Root	Zheng et al. (1988)
4	4, 4-Diethylcarbamoyl butyric acid	Root	Yang et al. (2008)
5	Diisobutyl methylmalonate	Root	Yang et al. (2008)
6	3,7,11-Dimethyl-1,6,10-dodecatriene-3-ol	Root	Yang et al. (2008)
7	3, 3-Dimethyl-hexane	Root	Yang et al. (2008)
8	Ethyl octadecanate	Fruit	Lu et al. (1998)
9	Ethyl palmitate	Fruit	Lu et al. (1998)
10	Linoleic acid	Flower bud	Li, et al. (1999)
11	Linolenic acid	Flower bud	Li et al. (1999)
12	Methyl caproate	Root	Zheng et al. (1988)
13	Methyl caprylatc	Root	Zheng et al. (1988)
14	Methyl decadienate	Root	Zheng et al. (1988)
15	Methyl 1,8-heneicosadienate	Fruit	Lu et al. (1998)
16	Methyl N-heptadecanoate	Root	Zheng et al. (1988)
17	Methyl heptylate	Root	Zheng et al. (1988)
18	Methyl 14-methyl-pentadecanate	Fruit	Lu et al. (1998)
19	Methyl n-octadecanoate	Root	Zheng et al. (1988)
20	Methyl 9, 12, 15-octadeca trienoate	Root	Zheng et al. (1988)
21	Methyl oleate	Root	Zheng et al. (1988)
22	Methyl palmitate	Root	Zheng et al. (1988)
23	Methyl palargonate	Root	Zheng et al. (1988)
24	Methyl n-pentadeeanoate	Root	Zheng et al. (1988)
25	Nonane-decanoic acid	Root	Yang et al. (2008)
26	Oleanolic acid	Flower bud	Chen and Cui (2002)
27	Palmitic acid	Root, flower bud	Chen and Cui (2002) Yang et al. (2008)
28	Stearic acid	Flower bud, fruit	Li et al. (1999) Lu et al. (1998)
29	2,4,7,9-Tetramethyl-5-decyne-4,7-diol	Root	Yang et al. (2008)

### Mineral Elements

PQR contains macroelement, including Ca, K, Na, Mg. The essential microelements include: V, Cr, Mn, Fe, Co, Ni, Cu, Zn, Se, Mo, Sn, etc. Rare earth elements include: Y, La, Ce, Pr, Nd, Sm, Eu, Gd, Tb, Dy, Ho, Er, Tm, Yb, Lu, etc. Toxic elements such As, Pb, Hg and Th radioactive elements also contain Li, Be, B, Al, Ga, Rb,As, Sr, Ag, Cd, Cs, Ba, Ti, Bi, U, I, P, Ti, Si and other elements (Liu et al., 1987; Zhang et al., 1987) (Zhang et al., 1988a; Feng et al., 2018). Zhang’s research showed that the content of mineral elements in the aboveground part (stem, leaf and seed) of PQR was higher than that in the underground part. And the content of mineral elements in the stems and leaves which had not been used as medicine in the past was much higher than that in the taproot. Moreover, various mineral elements in PQR can promote the growth of children and other therapeutic effects, on the other hand, they can play a role in disease prevention and health care.

In addition, PQR also contains vitamins A, B, B2, B6 (Chen and Cui, 2002), superoxide dismutase (SOD), peroxidase (POD), esterase (ES) (Wu and Lin, 1991), nitrate reductase (NR), catalase (CAT), polyphenol oxidase (PPO), ascorbate peroxidase (APX) (Wang S. et al., 2016), crude protein, crude fiber and other components (Sun and Liu, 1993)

## Physiochemical Composition

### Saponins

**FIGURE 2:**
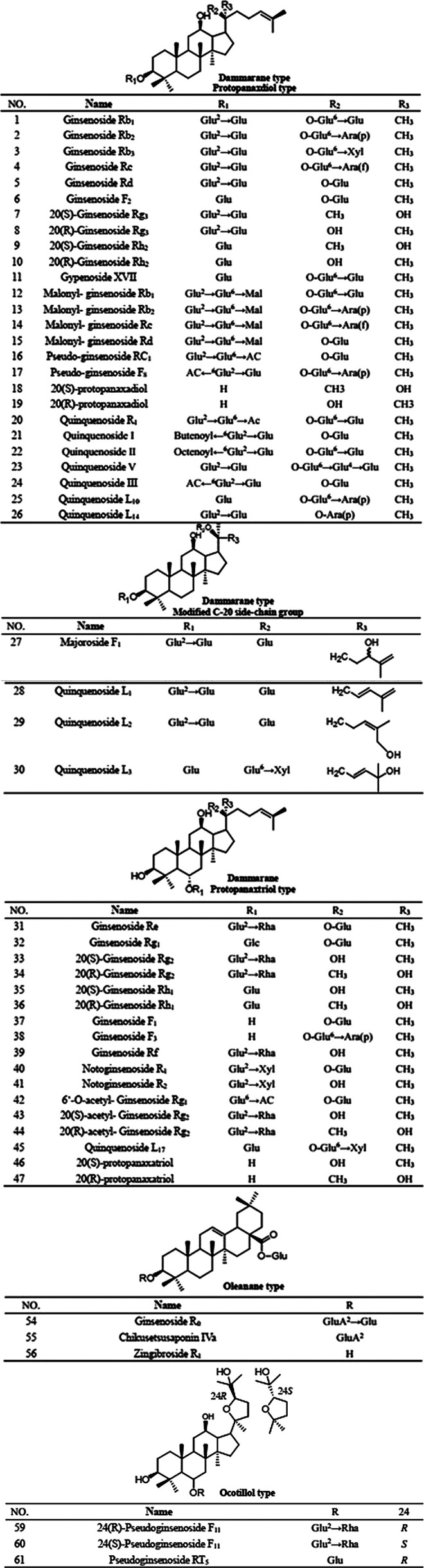
The structures of saponins compounds of panacis quinquefolii radix.

PQR mainly contains ginsenosides, which can be divided into three types according to their parent structure: dammarane, oleanane and oceanillol. Studies have confirmed that ginsenosides are contained in roots, stems, leaves, flowers, fruits and seeds of PQR. Now more than 70 ginsenosides have been isolated from different parts of PQR (as shown in Table 5; Figure 2). Meng et al. studies showed that the total saponins content in different parts of PQR was as follows: flower bud > flower stalk > pulp > root > stem and leaf, the highest content of saponins in flower bud was 14.87%, and the lowest was 5.79% in stem and leaf (Meng et al., 2000). Wang et al. showed that the stem, leaves and taproot of PQR contain saponins, and the content of ginsenosides in the stem was 1/3-1/2 of that in the taproot, while the content of ginsenosides in the leaves was 1.5–4 times of that in the taproot (Wang and Dong, 1995). In other words, because the leaves of PQR are not exploited and utilized, a lot of the medicinal ingredients are lost, which results in a great waste of resources. Future scholars can focus on the stems and leaves of PQR in order to make full use of its resources. As mentioned above, PQR is expensive and the resources are in short supply. If the root of PQR is used as medicine, abandoning the buds of stems and leaves will cause a great waste of resources. The above studies also showed that the content of saponins in the flower bud was the highest, followed by the fruit. In Pharmacopoeia, the plants with dried flower buds and fruits as medicinal parts were not rare. Future scholars can study the pharmacological effects or edible value of the flower buds and fruits of PQR. On the one hand, it can make full use of PQR resources and alleviate the pressure of supply exceeding demand of the market of PQR. On the other hand, it can develop new medicinal parts of PQR and enrich its clinical application and food health application.

**TABLE 5:** The saponins compounds of panacis quinquefolii radix.

Type	No	Name	Source	References
Dammarane	1	Ginsenoside Rb1	Root, stems, leaves, fruit, flower bud	Li et al. (1998), Meng et al. (2000),Guo et al. (2006), Wang et al. (2007)
	2	Ginsenoside Rb2	Root, stems, leaves, fruit, flower bud	Meng et al. (2000), Cao (2004), Wang et al. (2007)
	3	Ginsenoside Rb3	Root, stems, leaves, fruit, flower bud	Meng et al. (2000), Cao (2004), Jing and Zhao (2007), Wang et al. (2007)
	4	Ginsenoside Rc	Root, stems, leaves, fruit, flower bud	Li et al. (1997), Meng et al. (2000), Jing and Zhao (2007)
	5	Ginsenoside Rd	Root, stems, leaves, fruit, flower bud	Meng et al. (2000), Cao (2004), Jing and Zhao (2007)
	6	Ginsenoside F2	Root, stems, leaves3	Chen and Cui (2002), Cao (2004)
	7	20(S)-ginsenoside Rg3	Root, fruit, flower bud	Li et al. (1997), Li et al. (1998), Tang et al. (2000)
	8	20(R)-ginsenoside Rg3	Fruit, flower bud	Wang et al. (2007)
	9	20(S)-ginsenoside Rh2	Root, stems, leaves, fruit	Li et al. (1998), Cao (2004), Wang et al. (2007)
	10	20(R)-ginsenoside Rh2	Root, fruit	Li et al. (1997), Wang et al. (2007)
	11	Gypenoside XVII	Root, stems, leaves	Li et al. (1997)
	12	Malonyl- ginsenoside Rb1	Root, fruit	Li et al. (1998), Yu (1998)
	13	Malonyl- ginsenoside Rb2	Root	Wang et al. (2007)
	14	Malonyl- ginsenoside Rc	Root	Wang et al. (2007)
	15	Malonyl- ginsenoside Rd	Root	Wang et al. (2007)
	16	Pseudo-ginsenoside RC1	Root	Wang and Su (2011)
	17	Pseudo-ginsenoside F8	Root	Wang and Su (2011)
	18	20(S)-protopanaxadiol	Root	Wang and Su (2011)
	19	20(R)-protopanaxadiol	Root	Wang and Su (2011)
	20	Quinquenoside R1	Root, stems, leaves	Bao et al. (1998), Wang et al. (2007)
	21	Quinquenoside I	Root	Cao (2004)
	22	Quinquenoside II	Root	Cao (2004)
	23	Quinquenoside V	Root	Cao (2004)
	24	Quinquenoside III	Root	Wang et al. (2007)
	25	Quinquenoside L10	Root	Wang and Su (2011)
	26	Quinquenoside L14	Root	Wang and Su (2011)
	27	Majoroside F1	Stems, leaves	Li et al. (1997)
	28	Quinquenoside L1	Stems, leaves	Wang et al. (1997)
	29	Quinquenoside L2	Stems, leaves	Li et al. (1997)
	30	30Quinquenoside L3	Stems, leaves	Li et al. (1997)
	31	Ginsenoside Re	Root, stems, leaves, fruit, flower bud	Li et al. (1998), Meng et al. (2000), Guo et al. (2006)
	32	Ginsenoside Rg1	Root, stems, leaves, fruit, flower bud	Li et al. (1998), Meng et al. (2000), Cao (2004)
	33	20(S)-ginsenoside Rg2	Root, stems, leaves, fruit, flower bud	Li et al. (1997), Li et al. (1998), Tang et al. (2000)
	34	20(R)-ginsenoside Rg2	Fruit	Wang et al. (2007)
	35	20(S)-ginsenoside Rh1	Root, stems, leaves, fruit	Hao et al. (2000), Guo et al. (2006)
	36	20(R)-ginsenoside Rh1	Root, fruit	Guo et al. (2006), Wang et al. (2007)
	37	Ginsenoside F1	Root	Wang et al. (2007)
	38	Ginsenoside F3	Root, stems, leaves	Bao et al. (1998), Wang et al. (2007)
	39	Ginsenoside Rf	Root	Bao et al. (1998)
	40	Notoginsenoside R1	Root	Qu (2009)
	41	Notoginsenoside R2	Root	Qu (2009)
	42	6′-O-acetyl- ginsenoside Rg1	Root	Wang and Su (2011)
	43	20(S)-acetyl- ginsenoside Rg2	Root	Wang and Su (2011)
	44	20(R)-acetyl- ginsenoside Rg2	Root	Wang and Su (2011)
	45	Quinquenoside L17	Root	Wang and Su (2011)
	46	20(S)-protopanaxatriol	Root	Wang and Su (2011)
	47	20(R)-protopanaxatriol	Root	Wang and Su (2011)
	48	Quinquenoside F1	Root, fruit	Hao et al. (2000), Guo et al. (2006)
	49	Gypenoside IX	Stems, leaves	Li et al. (1997)
	50	Gypenoside XI	Root	Chen and Cui (2002)
	51	Gypenoside X	Root	Chen and Cui (2002)
	52	Gypenoside VII	Root	Chen and Cui (2002)
	53	Ginsenoside RA0	Root, stems, leaves	Bao et al. (1998), Wang et al. (2007)
Oleanane	54	Ginsenoside R0	Root, fruit	Wang and Su (2011)
	55	Chikusetsusaponin IVa	Root	Wang and Su (2011)
	56	Zingibroside R1	Root	Huang et al., (2017)
	57	Quinquenoside R3	Root	Li et al. (1997)
	58	Quinquenoside R4	Root	Li et al. (1997)
Ocotillol	59	24(R)-pseudoginsenoside F11	Root, stems, leaves, fruit, flower bud	Meng et al. (2000), Cao (2004), Jing and Zhao (2007)
	60	24(S)-pseudoginsenoside F11	Root, stems, leaves, fruit, flower bud	Meng et al. (2000), Cao (2004), Jing and Zhao (2007)
	61	Pseudoginsenoside RT5	Stems, leaves, fruit	Li et al. (1997), Wang et al. (2000)
Other	62	Quinquenoside Ⅳ	Root	Cao (2004)
	63	Ginsenoside Rg 6	Root	Guo et al. (2006)
	64	Linarionoside A	Stems, leaves	Cao (2004)
	65	Ginsenoside Rg8	Root	Wang et al. (2007)
	66	Quinquenoside F5ab	Fruit	Liu et al. (2010)
	67	Quinquenoside L10	Fruit	Liu et al. (2010)

### Volatile Oil

Shen et al. identified 37 compounds from the volatile oil of PQR cultivated in Jilin province, among which 26 sesquiterpenes accounted for 75% of the total volatile oil, the content of *trans*-*β*-farnesene was as high as 36%, and the content of bisabolene was as high as 10%, which were the main components of the volatile in PQR (Shen and Wang, 1991). Yang and Meng et al. isolated 47 and 39 volatile oil components from the root of PQR, which also indicated that the content of β-farnesene was the highest (Meng et al., 2001; Yanget al., 2008). Zheng et al. isolated and identified the volatile oil components of PQR cultivated in Heilongjiang province. The basic components contained ketones, alcohols, phenols and aldehydes. The strong acidic components were dominated by organic acids, and also contained aldehydes and ketones. The weak acidic components were mainly contain alkene, and also contained ketones and esters.The neutral components were dominated by esters and also contain alkenes and phenols (Zheng and Zhang, 1993).

### Polyacetylene

Polyacetylene is an unsaturated compound with a carbon-carbon triple bond. Polyacetylene is one of the active ingredients of PQR. So far, a total of 15 polyacetylene have been reported in PQR, such as acetylpan axydol, (6R, 7S)-6, 7-epoxyte-tradeca-1,3-diyne, falcalinol, ginsenoyne G, heptadeca-1,8-diene-4,6-diyne-3,10-diol, panaxydol, panaxytriol, panaquinquecols I1, panaquinquecols I2, panaquinquecols I3, PQ-1, PQ-2, PQ-3, PQ-7, PQ-1, PQ-8 (Fujimoto et al., 1991; Fujimoto et al., 1994; Satoh et al., 1997; Bao et al., 1998).

### Sterol

Sterol has many functions such as anti-tumor, promoting metabolism, regulating hormone level and preventing cardiovascular disease. At present, a variety of sterols have been isolated from PQR, daucosterol has been isolated from root and stem of PQR. β-sitosterol (Zhang, 1997), stigmasterol and stigmasterol-3,5-dien-7-one were obtained from the roots of PQR (Zhang et al., 1988b). Stigmast- 5- en- 3- ol was identified from the fruit of PQR (Wang et al., 2007).

### Flavone

Wei et al. isolated two flavonoid monomers, kaempferol and panasenoside, from the leaves of PQR. Meng et al. first discovered the existence of panasenoside in the root and fruit of PQR (Meng et al., 2002). And Liu (2008). isolated panasenoside II and trifolin from PQR. Flavonoid is one of the natural active ingredients which has been widely concerned in recent years. It has the functions of anti-oxidation and sterilization At present, there are few studies on the flavonoids in PQR, and it is hoped that future scholars could pay more attention to this compound with strong pharmacological activity.

### Other

In addition, PQR contains nucleoside compounds: deoxyuridine, deoxythymidine, deoxyadenosine and adenine. Lignan compounds such as pinoresinol and 4,4′, 8′ - trihydroxy-3,3′- dimethoxy-9′ – lignanolide. And Panaxynol compounds include panaxynol, panaxytriol, panaxynol A and panaxynol D. PQR also contains polyphenols compounds such as vanillic acid, p-coumaric acid, cinnamic acid (Jing et al., 2011), caffeic acid and chlorogenic acid (Wang et al., 2007).

With the deepening of the research on PQR, the ministry of health has approved it as a medicinal and food homologous product. The successful introduction and planting of PQR in China provides good materials for clinical, medical and other fields.

At present, the research on the chemical composition of PQR mainly focuses on the determination of its saponins, amino acids and trace elements.There are relatively few studies on the analysis of proteins, fatty acids and other organic compounds and their functions, which may be due to the complex components of proteins and polysaccharides, which are difficult to separate and purify. Moreover, in the process of extraction of PQR and the determination of substance content and types in the residue after production, it was found that a large amount of crude protein and crude polysaccharide could not be effectively used and could only be discarded with the discharge of waste residue. PQR as a high-grade health care products, the price is expensive, and the demand exceeds supply. According to the determination, the content of saponins and polysaccharides in the leaves of PQR is as high as more than 10%, which is twice as much as that in the root of PQR. In other words, because the leaves of *Panax quinquefolium* are not exploited and utilized, 2/3 of the medicinal ingredients are lost. And modern pharmacological studies have shown that PQR polysaccharides have good immunomodulatory and antioxidant activities. Due to the high price of PQR, future scholars can focus on the stems and leaves of PQR. Therefore, how to make full use of PQR remains to be further studied.

### Quality Control

With the increasing demand for PQR, PQR is in short supply and expensive. In order to seek excessive profits, the adulterants of PQR increase accordingly, and the phenomenon of shoddy products frequently occurs in the market. Moreover, plant resources are easily affected by different factors, such as provenance, growth environment, harvesting time, processing methods and storage conditions, which makes the quality of PQR from different regions uneven. In addition, PQR has complex chemical components and many chemical substances have pharmacological effects. The quality control of medicinal materials is the most problematic link in the production of medicinal materials. At present, the key problem in the quality evaluation of PQR is that the evaluation index is too one-sided and unitary.In Chinese pharmacopoeia (2020 edition), only Rg1, Re and Rb1 were used as the quality control indexes of PQR. However, medicine materials contains many kinds of ingredients, different ingredients work together, it is not scientific and objective to evaluate the quality of medicine materials only by using one or several effective components as indicators. Therefore, in order to ensure the safety and effectiveness of PQR, it is necessary to establish a rapid, accurate and sensitive detection method to simultaneously measure a variety of chemical components, evaluate the quality of medicinal materials, and improve the quality control methods.

Some qualitative and quantitative methods have been used to evaluate the quality of PQR. In the past, scholars mostly used thin-layer chromatography to qualitatively identify PQR (Liu Y. et al., 2016), or established fingerprint of PQR by high-performance liquid chromatography for the qualitative identification (Tang et al., 2015).In the past, colorimetry (Yin et al., 2010), near infrared spectroscopy (Huang et al., 2011) nuclear magnetic resonance (NMR) spectroscopy (Nabuurs et al., 2017) inductively coupled plasma method (Jiang et al., 2020)gas chromatography (Lin et al., 2018), high performance liquid chromatography in series with various detectors (Chen et al., 2020, Xiong et al., 2020), and immunoassay (Morinaga et al., 2010) were used for multi-component determination and quality control of PQR. However, there are some defects in the design of these methods.

These studies did not clarify the quality markers of PQR. They only determined and analyzed the saponins and mineral elements commonly existing in the PQR, and did not show that these components are the main active substances of PQR. The future research of quality control should consider integrating quality overall evaluation technology and Internet analysis technology. In order to promote the sustainable development of PQR industry, the pharmacological effects of active ingredients and the efficacy of Chinese medicine can be studied in an integrated way.

Secondly, previous methods lack of studies on other compounds in PQR except saponins, volatile substances and mineral elements. Chinese medicine has the characteristics of multi-component and multi-target joint effect. Only one or two kinds of compounds as the main research direction is far from meeting the needs of the development of traditional Chinese medicine. Besides, PQR, as a medicinal and food homologous product, should pay more attention to the study of the effective active components such as carbohydrate, fatty acid and amino acid in future quality control research.

Finally, most of the methods have some disadvantages, such as complex sample pretreatment, using toxic organic solvents, polluting the soil and not green enough. Future scholars should not only focus on the existing methods, but also try to adopt other methods to control the multi-component quality of PQR. For example, Hou et al. used UPLC-QDA method to analyze the spectral effect of Angelicae Pubescentis. Compared with other mass spectrometry methods, this method is cheaper and more cost-effective (Houet al., 2020).Yang et al. established the fingerprint of Angelica Pubescens by UPCC method, and analyzed the overall information of Angelica Pubescens. The method uses CO_2_ as mobile phase, which is environmentally friendly and green (Yang et al., 2020). At the same time, it is also hoped that with the rapid development of science and technology, future engineers will develop more and more inexpensive, efficient instruments and green reagents.

### Pharmacology

Modern pharmacological studies have shown that American ginseng has neuroprotective (Chanana and Kumar. 2016), anti-cardiovascular disease (Mehendale et al., 2006), anti-tumor (He et al., 2011), immunomodulatory (Qi et al., 2016), anti-hypertension (Wu et al., 2007), anti-diabetes (Sievenpiper et al., 2003), anti-oxidation (Hu and Kitts, 2001), anti-inflammatory (Xie et al., 2018), antibacterial, antiviral (Mousa, 2015), anti-radiation and other effects (Liao et al., 2019). These pharmacological effects are summarized in Table 6 and discussed in detail below.

**TABLE 6:** The pharmacological effects of panacis quinquefolii radix.

Effects	Extracts/compounds dose	Animal/cell line	Study design	Control	Mechanism/results	References
Neuroprotection	PQR extract 50, 100, and 200 mg/kg	Anxiety-like behavior in mice induced by sleep deprivation	*In vivo*	Picrotoxin Flumazenil Muscimol	PQR can antagonize anxiety-like behaviors, oxidative stress state, changes in mitochondrial enzyme complex activity, elevated serum corticosterone level and brain TNF-α level caused by 72-h sleep deprivation, as well as neuroinflammatory signs in discrete brain regions	Chanana and Kumar (2016)
	PQR extract 50, 100, and 200 mg/kg	Chronic unpredictable stress mice model	*In vivo*	l -NAME l -arginine	It plays a neuroprotective role by regulating the nitrergic signaling pathway	Rinwa and Kumar (2014)
	PQR saponin 0, 50, 100, 150, and 200 ng/ml	A rat spinal cord injury model	*In vitro*	Saline	It can improve cell damage, inhibit cell apoptosis, enhance neurite repair function, and reduce the expression of Bax and cleaved caspase-3, GRP78 and PDI.	Dou et al. (2018)
	PQR extract Rb1 1 mg/ml	TsA201 cell	*In virto*	Xylocaine	It has a neuroprotective effect by blocking sodium channels	(Liu et al., 2001)
	PF 11 4 and 8 mg/kg	Neurotoxicity model of mice induced by MA	*In vivo*	Saline	PF11 could up regulate the contents of dopamine (DA), 3,4-dihydroxyphenylacetic acid (DOPAC), isovanillic acid (HVA) and 5-hydroxyindoleacetic acid (5-HIAA) in mice brain	Wu et al. (2003)
	PF 11 1, 4, or 8 mg/kg	Mice behaviors induced by methamphetamine	*In vivo*	Saline	Inhibition of meth-induced hyperactivity, preference, and increased extracellular DA in the nucleus accumbens by modulating gaba neurons and opioid receptors	Fu et al. (2016)
	PF 11 3, 6, and 12 mg/kg	Parkinson’s disease model of rat induced by 6-hydroxydopamine	*In vivo*	Madopar	PF 11 could inhibit the formation of free radicals and stimulate the release of endogenous antioxidants	Wang et al. (2013)
	PF 11 0.32, 1.6, 8 mg/kg	Cognitive deficits model of mice induced by Aβ 1-42	*In vivo*	Saline	PF 11 can inhibit the expression of amyloid protein in the cortex and hippocampus, alleviate the effect of oxidative stress, and protect the function of neurons	Wang et al. (2013)
	Above ground part of PQR 25 and 50 mg/kg	Neurodegeneration model of rats induced by 3-nitropropionic acid	*In vivo*	Saline	PQR can significantly affect the 3-nitropropionic acid-induced motor injury and cell loss of the striatum, and completely avoid mortality	Lian et al. (2010)
	Rb1 5 mg/kg/day	Sprague–Dawley rats	*In vivo*	Saline	Ginsenoside Rb1 plays a role in regulating the mRNA expressions of ChAT, NGF and trkA in the brain of rats	Salim et al. (1997)
	PQR 30% ethanol extract	Brain injury and cognitive deficits model of mice induced by Aβ 1–42F3.ChAT human neural stem cells	*In vivo In virto*	Blank	PQR can improve acetylcholine level through ChAT gene expression and neuroprotection	Shin et al. (2016)
	Rb1, Rb3, Rd	Epilepsy induced by kainic acid, pilocarpine or pentylenetetrazole	*In vivo*	None	Significantly increases the incubation period of epilepsy	Lian et al. (2006)
Cardioprotection	PQR berries 70% ethanol extract 0.5–2.5 mg/ml	Chick embryo cardiomyocyte	*In virto*	Blank	PQR has a protective effect on myocardial oxidative stress	Mehendale et al. (2006)
	Rb1 0.01, 0.1, 1 μM	Chick embryo cardiomyocyte	*In virto*	Blank	Rb1 plays a cardioprotective role by weakening ROS activity and inhibiting JNK activation	Li et al. (2012)
	PQR root extract	Diabetic mouse model	*In vivo*	Citrate buffer	PQR prevents biochemical and functional changes in the heart by inhibiting oxidative stress	Sen et al. (2013b)
	Shenyuan powder 240, 320, 400 mg/kg/d	A model of acute myocardial infarction caused by coronary artery ligation	*In vivo*	Metoprolol Tartrate	Inhibition of apoptosis was used to improve acute myocardial infarction	Zhu ,et al. (2013)
	PQR extract 0.5, 2, 8 μg/ml	Neonatal cardiomyocytes	*In virto*	Blank	PQR protects the heart from ischemia-reperfusion injury by upregulation of eNOS.	Wu et al. (2011)
	PQR saponin 50 mg/L	Neonatal rat ventricular myocytes	*In virto*	Compound C	PQR saponins protect neonatal rat ventricular myocytes from hypoxia reperfusion injury by activating GLUT4-pfk2 and FAT/CD36-ACC2 pathways mediated by AMPK-inhibitor	Yu et al. (2020)
	PQR extract	Neonatal ventricular cardiomyocytes	*In virto*	Blank	PQR alleviates cardiac hypertrophy by blocking p115rhogef/rock-dependent p38 MAPK activation	Moeyet al. (2011)
	PQR root extract	RAW264.7 H9C2 cells	*In virto*	Neat maltodextrin	PQR regulates the expression of NF-κB mediated cytokines in macrophages by activating Nrf2 signaling pathway	Qu et al. (2015)
	PQR extract 10 μg/ml	Isoproterenol-induced myocardial hypertrophy in rat model neonatal ventricular myocytes	*In vivo In virto*	None	The cardioprotective effect of PQR is associated with phosphorylation of protein kinase A and camp-responsive element binding proteins	Tang et al. (2016)
	PF 11 3, 6 and 12 mg/kg	Rat model of coronary artery ligation	*In vivo*	Metoprolol tartrate	By reducing infarction area, improving cardiac function, reducing serum LEVELS of CK, AST, LDH, MDA and cTnT, increasing SOD activity, and inhibiting b1-AR expression, cardiac protection can be played	Zhang et al. (2017)
Anticancer	Rh2	HCT-116 human colorectal cancer cells	*In vitro In vivo*	HI-TOPK-032	Rh2 inhibits tumor cell growth and induces apoptosis by inhibiting PBK/TOPK activity	Yang, et al. (2016)
	PQR root 70% ethanol extract	HCT-116 human colorectal cancer cells	*In vitro*	5-FU	The 5-Fu-induced S phase retardation and the combination of 5-Fu and PQR increased G1 phase	Wan et al. (2018)
	PQR extract	High fat-diet Apc ^Min/+^ mice	*In vivo*	Blank	Colon cancer can be prevented by anti-inflammatory and antioxidant mechanisms	Xie et al. (2015)
	PQR extract 15 and 30 mg/kg/day	AOM/DSS mouse model	*In vivo*	Blank	Colon cancer is prevented by inhibiting inflammatory cytokines and restoring metabolomics and microbiotics	Wang et al. (2016)
	PQR root extract	MDA MB 231 and MCF7 cell lines	*In vitro*	PDBu	It plays an anti-breast cancer role by inhibiting the activation of COX-2 and NF-κB	Peralta et al. (2009)
	Rg3	HCT-116 and SW-480 colorectal cancer cells	*In vitro*	Blank	Rg3 inhibits colorectal neoplasms by downregulating the Wnt/ß-catenin signaling pathway	(He et al., 2011)
	PQR hexane extract	HCT 116 WT cells	*In vitro*	MirVana-miR-29b inhibitor	Inhibit the migration of colon cancer cells	Poudyal et al. (2013)
	PQR saponin	SW-480, HT-29, HCT-116 human colorectal cancer cells IEC-6 rat small intestine epithelial cells	*In vitro*	Blank	Anti-colon cancer function is performed by mediating the breakdown factor-related apoptosis-inducing ligand (TRAIL) pathway to regulate the interaction between p53 and DR4/DR5	Zhang et al. (2015)
	Aqueous extracts of roots of PQR	SMMC-7721 cells	*In vitro*	Blank	By promoting the apoptosis of liver cancer cells to play the role of anti-liver cancer	Qu et al. (2018)
	Compound K	HCT-116 and SW-480 colorectal cancer cells	*In vitro*	Blank	Compound K inhibited cell G1 phase and induced apoptosis obviously	Yuan (2012)
	Heat-processed PQR	Human gastric cancer AGS cells	*In vitro*	Paclitaxel	It can significantly reduce the proliferation of gastric cancer cells and induce the apoptosis of cancer cells	Park et al. (2014)
	Heat-processed PQR	Human breast cancer cell lines MCF-7 and MDA-MB-231 cell lines	*In vitro*	Blank	The heat treatment of PQR could significantly improve its anti-proliferative activity, and the inhibition effect was G1 phase	Wang et al. (2008)
Immunoregulation	PQR polysaccharide 1, 12.5, 25, 50 μg/ml	RAW 264.7 murine macrophage cells	*In vitro*	Lipopolysaccharide	It plays an immunomodulatory role by stimulating the expression of proinflammatory factors and NOS2	Ghosh et al. (2019)
	Aqueous extracts of roots of PQR	Human peripheral blood mononuclear cells	*In vitro*	None	It plays an immunomodulatory role by up-regulating proinflammatory factors and mediating MAPK (ERK-1/2), PI3K, P38 and NF-κB signaling pathways	Lemmon et al. (2012)
	SZC 1.3, 2.6, and 5.2 g raw material/kg body weight	ICR male mice	*In vivo*	Sterile water	The immune ability is enhanced by regulating the level of immune organs and antioxidant activity	Yu et al. (2016); Yu et al. (2017)
	AGNP 2, 10, 50, 100, 200 μg/ml	Peritoneal macrophages	*In vitro*	Lipopolysaccharide	Through the regulation of inflammatory factors, increase the phagocytosis of macrophages, promote the production of nitric oxide to play the role of immune regulation	Qi et al. (2016)
	PQR acidic polysaccharides	RAW264.7	*In vitro*	Blank	Immune regulation is achieved by stimulating macrophages to secrete proinflammatory cytokines	Wang et al. (2015a)
Antidiabetic effect	PQR root extract	Db/DB induced type II diabetic mice	*In vivo*	Blank	It plays a hypoglycemic role by increasing the levels of insulin, glycogen and HDL, and lowering the levels of blood glucose, triglyceride, total cholesterol and low-density lipoprotein	Yoo et al. (2012)
	PQR extract	Diet-induced obese mice	*In vivo*	Blank	PQR has anti-obesity effect by regulating glycolipid metabolism	Zhang et al. (2019)
	PQR ethanol extracts 200 mg/kg BW/day	Streptozotocin-induced type I diabetes C57BL/6 mice Db/DB induced type II diabetic mice	*In vivo*	None	PQR plays a hypoglycemic role by regulating body weight, plasma insulin, C-peptide, blood glucose and glycosylated hemoglobin levels in diabetic mice	Sen et al. (2013b)
	PQR root extracts 42.33, 84.66 and 169.33 mg/kg	A high-fat diet and alloxan induced hyperglycemia in rat models	*In vivo*	Acarbose	PQR plays a role by inhibiting α-amylase and α-glucosidase levels to reduce the expression of GLUT4 and PDK1	Kan et al. (2017)
	PQR berry extract 150 mg/kg PQR leaves extract 50, 150 mg/kg	Db/DB induced type II diabetic mice	*In vivo*	Blank	It works by improving the weight of diabetic mice	Xie et al. (2002), Xie et al. (2004)
	PQR extract 2.7 g/day of	Participants with type II diabetes	*In vivo*	Cornstarch Wheat bran	It plays a hypoglycemic role by reducing glycosylated hemoglobin and lipid concentration	Jenkins et al. (2018)
Antihypertensive effect	PQR extract	Hypertensive patients over 65 years of age	*In vivo*	Benzenesulfonate amlodipine	It plays an anti-hypertensive role by reducing systolic blood pressure, increasing diastolic blood pressure, lowering ginger pulse pressure, and regulating the expression of adhesion molecules	Yang (2015)
	PQR extract	Hypertension patients	*In vivo*	None	PQR works by reducing central systolic pressure, end-systolic pressure and area under the systolic/diastolic curve	Jovanovski et al. (2020)
Anti-oxidation effect	PQR berry extract0.1, 0.5, 10 μg Re 0.05, 0.1 μg	MIN-6cellline	*In vitro*	Blank	PQR and Re play a role by scavenging free radicals and protecting cells	Lin, et al. (2008)
	PDD-ginsenoside 20, 30 μg	NHDF cells	*In vitro*	Arbutin	PPD showed antioxidant effects by increasing TGF- B1 level, enhancing Nrf2 nuclear translocation, and limiting AP-1 and MAPK phosphorylation	Liu et al. (2019)
	PQR extract	H9C2 cell line	*In vitro*	None	PQR can induce Nrf2 antioxidant gene expression by activating Nrf2 pathway	Li et al. (2010)
	PQR extract	Hepa1c1c7 cells	*In vitro*	β-Napthoflavone	PQR exerts its antioxidant effect by scavenging free radicals and FRAP	Chen et al. (2010)
	PQR polysaccharide	RAW 264.7	*In vitro*	Blank	The crude polysaccharide of PQR plays an antioxidant role by scavenging free radicals	Yu et al. (2014)
	PQR methanolic extracts	T98G glioma cell line	*In vitro*	Blank	The antioxidant activity of PQR was improved by ABTS and FRAP	Kochan et al. (2019)
anti-inflammatory effect	PQR polysaccharide 25, 50 μg/ml	3T3-L1 preadipocytes	*In vitro*	Blank	PQR polysaccharide plays an anti-inflammatory role by regulating inflammatory factors such as IL-6, Ccl5, Nf-κb, TNF-α and TLR4 receptors	Wilson et al. (2013)
	PPQN 10, 50, 150 μg/ml	RAW264.7 macrophages	*In vitro*	Blank	PQR polysaccharide has therapeutic effects on inflammation by inhibiting the release of NO, TNF-α, IL-1β, IL-6	Wang et al. (2015a)
	PQR extract 50 mg/kg/day	Neonatal mouse cardiomyocyte	*In vitro*	Diphenyleneiodonium	It exerts anti-inflammatory effects by inhibiting the myocardial NOX2- ERk1/2 -TNF-α signaling pathway, inhibiting TNF-α expression	Wu et al. (2016)
	PQR saponin 50, 100, 150, 200 ng/ml	Chondrocytes	*In vitro*	Blank	PQR saponin play an anti-inflammatory role in resistance to interleukin-1 -mediated apoptosis by activating the NF -κB pathway	Xie et al. (2018)
Antibacterial effect	PQR extract	*Candida* albicans	*In vitro*	Blank	The expression of some inflammatory cytokine markers was significantly reduced	Trammell et al. (2012)
Antiviral effect	Polysaccharide from stem and leaf of PQR	Hep2 cell	*In vitro*	Ribavirin	It has a good inhibitory effect on respiratory syncytial virus	Feng et al. (2017)
	PQR extract	Outpatient	*In vivo*	Entecavir	It plays an anti-hepatitis B virus role by reducing ALT, AST and Tbil levels, and improving LIVER fibrosis indexes such as IV-C,PV-III, LN and HA	Chen et al. (2011)

### Centralsystem Protection

PQR can resist anxiety like behavior caused by 72-h sleep deprivation, oxidative stress state, changes in mitochondrial enzyme complex activity, elevated serum corticosterone level, increased brain TNF-α level, and neuroinflammatory like signs in discrete brain regions Pretreatment with GABA agonist could enhance the protective effect of PQR, and the protective effect is more significant than itself (*p* < 0.05) (Chanana and Kumar, 2016). PQR can significantly improve cognitive impairment caused by chronic unpredictable stress (CUS), regulate oxidative stress markers in the hippocampus, mitochondrial enzyme complex activity, and the level of proinflammatory cytokines (TNF-α), acetylcholinesterase, and serum corticosterone. l-arginine could reverse the protective effect of PQR. And l-NAME could enhance the function of PQR (Rinwa and Kumar, 2014). In addition, PQR could also be used as a drug for the treatment of acute central nervous system injury. Studies have shown that PQR could improve endoplasmic reticulum stress and neuronal cultural-related cell apoptosis after acute spinal cord injury induced by triglyceride (TG), and promote functional recovery after spinal cord injury (Dou et al., 2018). Other studies have shown that the extract of PQR enhances and reversibly blocks the Na^+^ channel in the way of concentration and voltage dependence. And PQR changed the voltage dependence of inactivation and delayed the recovery after inactivation to protect the ischemic nerve (Liu et al., 2001).

Studies have shown that pseudoginsenoside-F11 (PF11) in PQR may be a potential drug candidate for the prevention and treatment of neurologic disorders caused by methamphetamine (MA) abuse. PF11 could significantly shorten the resting time of mice induced by MA, (*p* < 0.05) and significantly shorten the incubation period of MA, (*p* < 0.05) antagonize the decrease of dopamine (DA), 3,4-dihydroxyphenylacetic acid (DOPAC), homovanillic acid (HVA) and 5-hydroxyindoleacetic acid (5-HIAA) induced by MA (Wu et al., 2003). In addition, PF11 can inhibit methamphetamine induced hyperactivity, preference and the increase of extracellular DA by regulating GABA neurons and opioid receptors. It has effective neuroprotection, promotes learning and memory, and antagonizes the pharmacological effects of morphine (Fu et al., 2016). Wang et al. showed that PF11 in PQR could significantly improve the motor ability, motor balance and coordination ability of Parkinson rats. PF11 could also increase the expression of tyrosine hydroxylase (TH) in substantia nigra and the content of extracellular DA in striatum, decrease the levels of extracellular hydroxyl radical (∙OH), 2,3 - and 2,5-dihydroxybenzoic acid (2,3 - and 2,5-DHBA), and increase the level of extracellular ascorbic acid (AA) (Wang J. Y. et al., 2013). Other studies have shown that PF11 could significantly inhibit the expression of amyloid protein (APP) and Aβ 1-42 in the cortex and hippocampus, restore the activities of superoxide dismutase (SOD) and glutathione peroxidase (GSH-PX), and reduce the generation of malondialdehyde (MDA) in the cortex. And the expressions of JNK2, p53, and cleaved caspase 3 in the hippocampus were down-regulated by PF11. These results suggest that PF11 may be a potential drug for the treatment of AD (Wanget al., 2013).

The root of PQR has no protective effect on 3-nitropropionic acid (3-NP)-induced neurodegeneration, but the stems and leaves of its aerial part could reduce the volume of striatal lesions induced by 3-NP. The main reason is that the aerial part of PQR contains a higher level of ginsenoside, among which Rb1, Rb3 and Rd play a key role. Since the neurodegeneration induced by 3-NP is similar to the neuron loss in Huntington’s disease, PQR may have clinical application value in the prevention of neurodegeneration in Huntington’s disease or other neurological diseases (Lian et al., 2010). Chen et al. showed that Rb1, Rg1 and other important ginsenosides in PQR are well-known components to improve human cognitive function (Chen and Hui, 2012). *In vivo* studies have shown that it may play a role by increasing the expression of choline acetyltransferase and trkA mRNA in the basal forebrain and the expression of nerve growth factor mRNA in the hippocampus through the cholinergic system (Salim et al., 1997). *In vitro* studies have shown that PQR could protect against F3 cytotoxicity induced by Aβ 1-42 by enhancing the expression of CHAT gene. It can also restore the cognitive function of mice by regulating the concentration of microtubule-associated proteins 2, synaptophysin and acetylcholine in the brain (Shin et al., 2016). Shi’s study showed that daily oral consumption of PQR could enhance the neurocognitive function of aging mice, which may be related to the up-regulation of insulin and adrenaline gene expression in the brain (Shi et al., 2012). Moreover, 52 healthy volunteers were tested, and according to the double-blind, placebo-controlled, balanced, and cross-design principles, the study showed that PQR significantly improved working memory in middle-aged people (Ossoukhova et al., 2015). In addition, Rb1,Rb3 and Rd in PQR have a good neuroprotective effect on the epilepsy induced by heneic acid, pilocarpine or pentaerythrazol (Lian et al., 2006).

The above studies have shown that PQR has a good neuroprotective effect on neurological disorders such as Huntington’s disease, epilepsy, acute central nervous system injury, neurodegenerative and other diseases. However, there are common problems in the above experiments, such as the lack of a comparative study between positive control drugs and PQR, and the study only on related biochemical factors, and the lack of in-depth study on the target pathways, target proteins and relevant neuroprotective mechanisms. Previous literature indicated that rapamycin, sodium valproate, donepezil hydrochloride and other drugs could be used as positive drugs to explore neuroprotective effects. ALOX5, ADORA2A, CDK2, GSK3B and TLR4/JNK/caspase-3, AMPK/Mtor/ULK1 are the key targets and pathways of neuroprotective effect. Whether future scholars can use this as a starting point to explore the in-depth mechanism of the neuroprotective effect of PQR. In addition, attention to insulin/IGF signaling, mitochondrial function and glucose utilization in neurodegenerative models may contribute to a better understanding of diseases such as Alzheimer’s disease.

### Cardioprotective Effect

Previous studies have shown that H2O2 is an important reactive oxygen species (ROS) which could induce oxidative damage through the interaction with iron. The berry extract and ginsenoside Re of PQR have high cardioprotective effects against oxidative stress. Phenolic acids such as caffeic acid and chlorogenic acid in PQR fruit could up-regulate the ability of myocardial cells to metabolize H_2_O_2_ in a dose-dependent manner and reduce the oxidative damage of cardiomyocytes induced by H_2_O_2_. It has protective effect on myocardial oxidative stress. However, the specific mechanism is not clarified (Mehendale et al., 2006). Rb1 has the activity of scavenging DPPH and hydroxyl radical, and can inhibit the increase of JNK phosphorylation (*p* < 0.01), reduce the expression of P-JNK, and improve the cell survival rate after H_2_O_2_ exposure to play a protective role on myocardial oxidative stress (Li et al., 2012). PQR could also significantly inhibit the upregulation of extracellular matrix protein and vasoactive factor caused by oxidative stress, and reduce the mRNA expression of cardiac natriuretic factor and brain natriuretic factor, so as to prevent the biochemical and functional changes of heart caused by diabetes (Sen et al., 2013b). Zhu et al. have shown that *Panax quinquefolium* can significantly inhibit oxidative stress, balance the ratio of Bcl-2/Bax, and inhibit the activation of Caspase-3 (Zhu et al., 2013). In addition, it could also significantly increase the phosphorylation of Akt and eNOS protein levels in ischemia-reperfusion myocardial cells, and significantly reduce the infarct area and myocardial cell apoptosis in mice to improve myocardial infarction (Wu et al., 2011). And PQR saponin could significantly increase the survival rate of neonatal rat ventricular myocytes induced by hypoxia reperfusion, down regulate lactate dehydrogenase (LDH) leakage, slow down early cell apoptosis, increase energy production (*p* < 0.05), and increase the expression of glucose transporter 4 (GLUT4) and fatty acid translocation enzyme/cluster 36 differentiation (FAT/CD36) on cell membrane (*p* < 0.05) (Yu et al., 2020).

As a potential dietary supplement, PQR has been proved to be effective in preventing cardiomyocyte hypertrophy and heart failure. It could reduce the occurrence of myocardial hypertrophy by blocking leptin stimulating RhoA/ROCK and significantly reducing the expression of p115RhoGEF gene and protein (Moey et al., 2011). It also activates Nrf2 signaling pathway by inhibiting Kelch-like ECH-associated protein (Keap) 1, and significantly inhibits inflammatory macrophage-mediated cardiac cell death and hypertrophy to play a cardiac protective role (Qu et al., 2015). PQR could inhibit the harmful effects of adrenaline agonist isoproterenol on cardiac hypertrophy and function. *In vivo* experiments, PQR could attenuate the effect of isoproterenol on left ventricular function, increase heart quality, and decrease the up-regulation of fetal gene expression. *In vitro* studies, PQR completely inhibited isoprenaline induced hypertrophy on the surface area of myocytes (Tang et al., 2016). In addition, PF11, the characteristic component of PQR, could reduce the levels of MDA, aspartate aminotransferase (AST), creatine kinase (CK), LDH, cardiac troponin T (cTnT), increase the activity of SOD, and significantly improve cardiac function and reduce the area of myocardial infarction. PF11 could be used as a natural anti-myocardial ischemia drug to improve cardiac function (Zhang et al., 2017).

In summary, PQR has anti-myocardial ischemia, anti-myocardial infarction, anti-atherosclerosis and other effects. In recent years, more and more studies have preliminarily confirmed the effect of PQR on myocardial ischemia-reperfusion injury. In addition, PQR also has the function of anti-arrhythmia, protecting the damaged myocardium and anti-hemorrhagic shock. Although the current research on heart protection of PQR is more and more in-depth, however, there are still some problems to be further explored. For example, the key components that play a cardiac protective effect on PQR are mainly concentrated in the PQR crude extract and PF11, and there is a lack of research on the mechanism of effect on other components. Studies have shown that panaxadiol saponins and panaxatriol saponins also have cardioprotective effects (Wang and Shi, 2011). Then, apoptosis is the key link of myocardial injury. In addition to the known mitochondrial apoptosis and death receptor pathway (Liu et al., 2010), whether there are other apoptotic pathways involved in cardiomyocyte apoptosis, such as the endoplasmic reticulum associated apoptosis pathway located upstream of mitochondrial apoptosis and death receptor pathway. These problems can be explored in future studies.

### Anti-Tumor Effect

After cardiovascular and cerebrovascular diseases become the first major killer of human health, the incidence rate of tumors is increasing, and has become the second major killer of human life and health, and the incidence rate is increasing year by year.At the same time, more and more attention has been paid to the search for new anticancer drugs with high selectivity, strong activity, little toxic and side effects and wide application from medicinal and food homologous plants. PQR is a medicinal and food homologous plant, widely used as dietary additives, has many benefits to human health, can effectively prevent and treat cancer diseases (Yu et al., 2015). Studies have shown that the anticancer active ingredients in PQR can be used as anticancer drugs alone (Yang et al., 2016), and can also be used in combination with other chemotherapy drugs to improve the efficacy or reduce the toxic and side effects (Wan et al., 2018).

Inflammation or excessive intake of animal fat are often risk factors for colorectal cancer. The preventive effect of PQR on cancer is related to the reduction of damaged amino acid, organic acid, fatty acid and carbohydrate metabolism. The metabolites that PQR can significantly change include arachidonic acid, linolenic acid, glutamic acid, docosahexaenoic acid, tryptophan and fructose, all of which are related to inflammation and oxidation (Xie et al., 2015). Oral administration of PQR could significantly inhibit the increase of IL-1 α, IL-1 β, IL-6, G-CSF, GM-CSF and other inflammatory cytokines in inflammatory mice, and inhibit the obvious changes of intestinal microbial community to prevent colorectal cancer (Wang C. Z. et al., 2016). In addition, PQR can inhibit COX-2 and NF-κB and reduce the proliferation of human breast cancer cells through anti-inflammatory effects (Peralta et al., 2009).

He et al. showed that Rg 3 could inhibite colorectal cancer by down-regulating the Wnt/-catenin signaling pathway (He et al., 2011). Poudyal study showed that PQR could induce the expression of miR-29b with matrix metalloproteinase-2 (MMP-2) as a target, thus inhibiting the migration of colon cancer cells (Poudyal et al., 2013). And protopanaxadiol, as a promising colon cancer inhibitor, it plays an anti colon cancer effect by mediating the interaction between p53 and DR4/DR5 by mediating the necrosis factor-related apoptosis-inducing ligand (TRAIL) pathway (Zhang et al., 2015). *In vitro* studies showed that ginsenoside Rh2 significantly induced colon cancer cell death by regulating phosphorylation levels of protein kinases 1/2 (ERK1/2) and H3. *In vivo* studies showed that ginsenoside Rh2 could inhibite the growth of tumor cells (Yang et al., 2016). In addition, the water extract of PQR root could play an anti hepatoma effect by promoting the apoptosis of SMMC-7721 cells (Qu et al., 2018).

Intestinal bacteria are fully involved in ginsenoside metabolism, and after oral administration of PQR, intestinal bacteria can gradually hydrolyze the sugar chain of ginsenoside. Under the action of intestinal bacteria, ginsenosides Rb1, Rc and Rd were first transformed into ginsenoside F2 and then converted into compound K (as shown in Figure 2). Therefore, it is not the ginsenoside Rb1, the main component of PQR, that plays an anti-cancer role, but the intestinal bacterial metabolite Compound K has significant inhibitory activity on the growth of colorectal cancer cells (Yuan, 2012). In addition, in the treatment of colorectal cancer, when 5-FU combined with PQR, the cell proliferation inhibition rate was significantly increased. The ability of PQR to enhance the anticancer effect of 5-FU was related to apoptosis (Wan et al., 2018). PQR could significantly enhance its anti-proliferation effect on a variety of cancer cells after being processed at high temperature, because the structure and composition of saponins were changed at high temperature, and more low-polarity saponins were produced, such as ginsenosides Rg3, 20R-Rg3, Rk1 and Rg5, etc. These components can enhance the enzyme activity of caspase-3 and caspase-9, significantly reduce the proliferation of gastric cancer cells and induce the apoptosis of cancer cells (Park et al., 2014). Other studies have shown that the contents of ginsenoside Rb1, Re, Rc and Rd are reduced, the contents of Rg2 and Rg3 are increased after processing at high temperature, the expressions of cyclin A and cyclin D1 are significantly reduced, and the antiproliferative activity of PQR is significantly improved (Wang et al., 2008). In addition, PQR can significantly reduce the nausea and vomiting caused by cisplatin without affecting its anticancer properties. And studies have shown that the PQR extract can significantly increase NK cells and prolong the life span of leukemia mice in a dose-specific manner (Miller et al., 2011).

The above studies show that PQR has significant effect in the prevention and treatment of colorectal cancer, gastric cancer, liver cancer, leukemia and so on. In addition to its potential anticancer effect, PQR is often used as an adjuvant drug in cancer treatment to alleviate the toxic and side effects caused by chemotherapy drugs. However, the above studies are lack of research on monomer compounds, as well as the validation of *in vivo* experiments and clinical studies. It is difficult to provide scientific basis for clinical development of reasonable and reliable new anticancer drugs. In the future, scholars can further study the anticancer effect and mechanism of compounds such as Rg2, Rg3 and intestinal metabolite compound K in PQR, and add corresponding animal *in vivo* and clinical trials to verify their efficacy. It is believed that based on clinical practice, combined with modern pharmaceutical theories and technologies, the development and application of anticancer TCM will make new and greater progress.

### Immunomodulatory Effects

It is understood that polysaccharides in PQR could stimulate the immune system. PQR polysaccharide is a weaker immune stimulant than lipopolysaccharide, which has the characteristics of immune regulation. Its main action pathway is to promote cell proliferation and regulate the expression of proinflammatory mediators IFN-g, IL-23A, IL-6, TNF - α, MCP-1 and GM-CSF. Stimulation of NOS2 gene expression leads to increased levels of iNOS and downstream the level of NO. Furthermore, studies have shown that its immunoregulatory effects are also related to MAPK (ERK-1/2), PI3K, P38 and NF-κB pathways. And PQR could also be used as an adjuvant or as a nutritional drug, or in combination with other therapies (Lemmon et al., 2012; Ghosh et al., 2019). The health tea containing PQR and PQR polysaccharide can significantly improve the spleen, thymus index and T lymphocyte proliferation ability of immune mice, and improve the NK cell activity and hemolytic activity to improve the immune ability of mice. In addition, the activities of antioxidant enzymes (superoxide dismutase, catalase and glutathione peroxidase) which inhibit oxidative stress are also one of the functions of enhancing immune function (Yu et al., 2016; Yu et al., 2017). A new protein (AGNP) isolated from Panaxanthus quinquefolium can also significantly increase the phagocytosis of macrophages, promote the production of nitric oxide, promote the inactivation of TNF-α and the production of IL-6 to play an immunomodulatory role (Qi et al., 2016).

The above studies showed that PQR polysaccharide was the most effective material basis for immune regulation in PQR. The immunoregulation mechanism is mainly to regulate the expression of pro-inflammatory factors and the level of immune organs. And the dosage forms of PQR immunomodulator are mainly granule wrapped by nanometer biomaterials (Akhter et al., 2019). However, the present study has several limitations, such as the lack of research on the optimal extraction technology of polysaccharide compounds from PQR and the lack of research on the optimal effective dose of immune regulation of PQR. Because the content of polysaccharides extracted by different extraction methods is different, it is difficult to have an accurate index to measure the clinical dosage. Secondly, the mechanism of immunoregulation of PQR only stays at the level of inflammatory factors and immune organs. Whether it can exert a pharmacological effects in other ways remains to be further studied by future scholars. Finally, there are only studies on the gastrointestinal permeability, dissolution rate and other factors of PQR polysaccharide encapsulated with nano biomaterials. In the future, scholars can also consider developing cheaper, environmentally friendly, safe and effective materials to replace it, and develop more new dosage forms of Chinese medicine to facilitate clinical application.

### Antidiabetic Effect

Diabetes has become a worldwide epidemic disease, and is a chronic progressive disease. The main drugs for the treatment of diabetes include sulfonylureas, biguanides, insulin, etc. (Liu et al., 2018). However, these drugs have a lot of side effects, which often require combined medication and poor patient compliance. In recent years, more and more people began to use dietary supplements and herbal supplements. Both clinical and experimental studies have shown that PQR, as a food supplement, has significant and well-tolerated effects in regulating glycolipid metabolism, anti-diabetes and obesity.

Studies have shown that PQR can improve cellular immune response and mitochondrial function, increase insulin production, reduce pancreatic cell death, and improve postprandial blood glucose in patients with type II diabetes mellitus. However, the active components of PQR to improve the function of pancreatic cells have not been found (Wu et al., 2007). Arginine, cinnamic acid, ferulic acid and other bioactive substances in the root of PQR can increase the levels of insulin, glycogen and high-density lipoprotein (HDL) in the mice with type II diabetes, and meanwhile reduce the levels of blood glucose, triglyceride, total cholesterol and low-density lipoprotein (LDL) to play a hypoglycemic role (Yoo et al., 2012). Ginsenosides Rb 1, Rg1, Rb2, Rc may also play a role in hypoglycemic effect (Sievenpiper et al., 2003). In addition, protopanaxdiol (PDG) and Protopanaxatriol (PTG) types of ginsenosides in PQR leaves can prevent and treat obesity, fatty liver and hypertriglyceridemia in mice fed with high-fat diet (Liu et al., 2010). The mechanism may be mainly through the regulation of linoleic acid metabolism, cysteine and methionine metabolism, and the biosynthesis of unsaturated fatty acids (Zhang et al., 2019). PQR could significantly reduced body weight and plasma insulin and C-peptide levels of type II diabetic animals, while increased body weight and plasma insulin and C-peptide levels of type I diabetic animals. However, both of them could decrease the blood glucose and glycosylated hemoglobin levels and increase the area of islets. The mechanism of action may be to promote the regeneration of B cells, leading to increased insulin secretion. In addition, the effect of PQR on weight may also be one of the mechanisms of hypoglycemia in type II diabetes mellitus (Sen et al., 2013a). PQR could also dose-dependent inhibit α-amylase (IC_50_ = 73.2 μg/ml) and α-glucosidase (IC_50_ = 111.8 ng/ml), improve insulin sensitivity and glucose uptake in human fat cells, improve fasting glucose level, and reduce the expression of GLUT4 and PDK1 in fatty tissue induced by high-fat diet and alloxan (Kan et al., 2017). And PQR berry and leaf extract could also reduce the weight of ob/ob mice, with anti-hyperglycemia and anti-obesity activity, and have important clinical significance for the prevention and treatment of type II diabetes (Xie et al., 2002; Xie et al., 2004).

Traditional Chinese medicine in the treatment of diabetes can prevent some complications, but also significantly reduce the side effects of western medicine.Studies show that Konjac-mannan (KJM) can regulate the absorption rate of nutrients in the small intestine, and PQR has post-absorptive effects. Therefore, it can be seen that KJM plays a role of hypoglycemic effect by increasing insulin sensitivity, while PQR may play a role by increasing insulin secretion, which plays a different and complementary role when combined PQR with KJM (Vuksan et al., 2001). And PQR combined with konjac-based fiber blend could reduce glycosylated hemoglobin and lipid concentrations in patients with type II diabetes, improve the effectiveness of routine treatment (Jenkins et al., 2018). Furthermore, PQR has a good preventive effect on diabetic complications, such as kidney injury, arteriosclerosis, retinopathy, etc.

The above studies show that PQR has a good therapeutic effect on type II diabetes. The hypoglycemic activity of PQR is mostly studied by animal *in vivo* experiments and clinical double-blind control. In the future, more large sample size and longer-term controlled trials are needed to provide reliable data support for the wide application of PQR. Moreover, the composition and content of active ingredients in PQR are complex, which are often caused by varieties, medicinal parts (roots, leaves, fruits), extraction methods, harvest years, growing places and so on. Therefore, although the international community has made positive comments on the role of PQR in the treatment of diabetes and thinks that it is likely to be widely used in the future, but it is pointed out that the current PQR as a drug has not been standardized and can not be widely used. In the future, it is necessary to conduct in-depth study on different effective parts and monomer components of PQR, and to clarify the exact components and main mechanism of its anti diabetes and obesity, so as to reduce the risk of drug use in clinical patients.

### Antihypertensive Effect

At present, the number of hypertension patients in China has exceeded 330 million, and the antihypertensive drugs currently used in reducing systolic blood pressure are often accompanied by the decline of diastolic blood pressure. Some patients often cause angina pectoris or even myocardial infarction or ischemic stroke because of low diastolic blood pressure.

Some research show that PQR combined with antihypertensive drugs can reduce systolic blood pressure, increase diastolic blood pressure, and decrease pulse pressure significantly. It can inhibit inflammatory reaction by regulating the expression of adhesion molecules and reducing the levels of sICAM-1 and sE-selectin, so as to protect and repair vascular endothelial cells and resist vascular sclerosis (Yang, 2015). PQR, rich in Ginsenoside Rg3, can improve central systolic blood pressure and pulse wave components, reduce late systolic blood pressure and myocardial oxygen consumption, and reduce peripheral wave reflex to play a role in lowering blood pressure (Jovanovski et al., 2020). However, although some studies have shown that PQR has a significant effect on the treatment of hypertension, there is a lack of in-depth research on its related mechanism.The pathogenesis of hypertension is very complex, and regulating inflammatory response may be only one of the important links. PQR may also play its role by regulating immunity, regulating the levels of endogenous substances Na^+^ and Ca_2_^+^ in vascular wall cells, and regulating cardiomyocytes, etc., which need further study and discussion. Moreover, most of the antihypertensive models of PQR are clinical subjects with small sample size, large individual differences, single pathological models and various external influencing factors. In the future, scholars can use chemical reagents to induce different types of hypertension animal models, study the mechanism of action of PQR one by one, so as to improve the clinical treatment of various types of hypertension with PQR.

### Antioxidation Effect

Studies have shown that PQR can delay the time of lipid peroxidation by scavenging DPPH free radicals, peroxidation (LOO•) and hydroxyl (•OH) free radicals, and protect human low-density lipoprotein from the influence of Fe_2_^+^, Cu_2_^+^ and Fe_3_^+^ mediated oxidation. It also has a similar protective effect on superhelical DNA breaking induced by hydrogen peroxide free radicals. And the antioxidant activity may be related to the content of Ginsenoside Rb1/Rb2 (Hu and Kitts, 2001). PQR berry extract and ginsenoside Re can reduce the oxidative damage of pancreatic cells induced by hydrogen peroxide, which can directly scavenge free radicals, prevent pancreatic cells from oxidative stress damage, and better regulate the blood glucose level in human body (Lin et al., 2008). PDD-ginsenoside is isolated from PQR and can be used as a potential natural antioxidant. PDD-ginsenoside can block the production of ROS under ultraviolet exposure, limit the production of MMP-1, and promote the synthesis of type I procollagen. Inhibition of vascular endothelial growth factor and TNF-α by NFAT signaling pathway. PDD-ginsenoside showed antioxidant effects by increasing TGF-β1 level, enhancing Nrf2 nuclear translocation, and limiting AP-1 and MAPK phosphorylation. PDD ginsenoside could inhibit melanin secretion and tyrosinase activity, reduce the content of Melan-A and zebrafish embryos, protect skin from light damage caused by ultraviolet rays, and prevent skin pigmentation (Liu et al., 2019). Other studies have shown that PQR could induce Nrf2 antioxidant gene expression by activating Nrf2 pathway (Li et al., 2010). In addition, ginsengsaponin g1 has a high oxygen free radical absorption capacity (Chen et al., 2010). And PQR crude polysaccharide also has obvious free radical scavenging effect (Yu et al., 2014). And the antioxidant activities of ginsenosides and phenolic acids in PQR could be improved by ABTS (scaling of free radicals) and ferric reducing antioxidant power (FRAP) (Kochan et al., 2019).

The above studies showed that PQR has good antioxidant activity. However, the current experimental research is limited to the cell level *in vitro*. In the future, scholars can consider using animal experiments and epidemiological methods of the relationship between antioxidant capacity in human serum and certain diseases to study the antioxidant mechanism and capacity of PQR *in vivo*. Moreover, at present, few studies have explored the monomer compounds that play the antioxidant role in PQR. In the future, scholars can use the spectrum–effect relationship method to screen the active substances that play the antioxidant role in PQR. Synthetic antioxidants often have some toxic and side effects, and PQR is expected to become a low-toxic and highly effective food antioxidants. In addition, the study showed that phenolic acids and flavonoids had strong antioxidant effect, phenolic acids and flavonoids accounted for a high proportion in the leaves and fruits of PQR, but the root was the medicinal part specified in the Pharmacopoeia. In the future, scholars can conduct in-depth study on the pharmacological effects of the stems, leaves and fruits of PQR, so as to provide the basis for the rational development and utilization of PQR.

### Anti-Inflammatory Effect

PQR is a popular natural health care product with anti-inflammatory properties. Wilson’s study showed that PQR polysaccharide could play an anti-inflammatory effect by regulating IL-6, NF-κB (Nuclear factor-Kappab), Ccl5 [Chemokine (C-C motif) ligand 5], TNF-α and other inflammatory factors, and TLR4 receptor could reduce the up-regulation of inflammatory gene expression, and also plays an important role in the regulation of inflammation (Wilson et al., 2013). Subsequently, it was found that the neutral polysaccharide PPQN in PQR has a therapeutic effect on inflammation and inflammation-related diseases by inhibiting the release of inflammatory mediators (NO) and cytokines (TNF-α, IL-1β, IL-6) (Wang et al., 2015b). The water extract of PQR could significantly inhibit the expression of NOX2, superoxide generation, ERK1/2 phosphorylation and TNF-α in the heart during endotoxemia by inhibiting the signaling pathway of NOX2-ERK1/2-TNF-α, and improve the level of inflammatory factors to improve cardiac function (Wu et al., 2016). PQR saponins could inhibit endoplasmic reticulum stress and associated chondrocytes' inflammatory response by activating the NF-κB pathway, and protect chondrocytes against triglycerides-induced endoplasmic reticulum stress and interleukin-1 -mediated apoptosis, thereby eliminating cartilage degeneration in rats (Xie et al., 2018).

PQR has good therapeutic effect on various diseases caused by inflammation, including chondroarthritis, endotoxemia, enteritis, myocardial injury and other diseases. However, the research on the anti-inflammatory mechanism of PQR is limited to the level of inflammatory factors, lacking the in-depth exploration of its anti-inflammatory mechanism. And there is a lack of research on effective anti-inflammatory ingredients. Future scholars can establish the network pharmacology of the anti-inflammatory effects of PQR to screen its effective anti-inflammatory components, potential targets and action pathways, and verify it through animal and clinical studies, so as to improve the anti-inflammatory mechanism of PQR.

### Antibacterial and Antiviral Effect

The active components of PQR, such as ginsenoside, polysaccharide, volatile oil, protein and panaxatriol, have antibacterial activities against pseudomonas aeruginosa, helicobacter pylori, staphylococcus aureus, escherichia coli, propionibacterium spinosum, *Candida albicans* and *Fusarium oxysporum*. The antibacterial mechanism includes 1) Affecting the formation of biofilm and destroying the mature biofilm. 2) Induction of apoptosis. 3) Stimulate the immune system and reduce microbial-induced cell apoptosis, inflammation and DNA damage. 4) Significantly reduced the expression of inflammatory factors. 5) Inhibition of antibiotic efflux (Wang and Ng, 2000; Trammell et al., 2012; Wang L. et al., 2020).

Frequent changes in the antigen structure of respiratory viruses have hindered the development of new vaccines. Society uses traditional herbal medicine to prevent and treat viral respiratory diseases. Studies have shown that the mechanism of PQR in preventing or treating influenza and influenza-like diseases may be related to the presence of substances that prevent the virus from replicating in human body (Mousa, 2015). Polysaccharide extracts from stem and leaf of PQR had good inhibitory effect on respiratory syncytial virus, and its therapeutic index TI was 32.57 (Feng et al., 2017). And PQR combined with bezoar has a good inhibitory effect on hepatitis B virus, could significantly reduce the levels of ALT, AST, Tbil, improveIV-C, PV-III, LN, HA and other indicators of liver fibrosis, improve liver function of patients, and have no side effects. It provides a new choice for clinical treatment of hepatitis B, which is worthy of further promotion and research (Chen et al., 2011). In addition, PQR has protective effects on both viral myocarditis and herpes simplex virus infected cells.

The above studies have shown that PQR has antibacterial and antiviral effects. Due to its unique natural, edible, low toxicity and high efficiency characteristics, as well as broad-spectrum antibacterial and antiviral activity and immune-enhancing effects, it is expected to become an important screening target for new antibacterial and antiviral drugs. The corona virus disease 2019 (COVID-19) outbreak at the end of 2019 has yet to develop a suitable vaccine or specific medicine. Studies have shown that traditional herbal medicine has a significant effect in the prevention and treatment of viral respiratory diseases. Ginseng Toxin-Vanquishing Powder (人身败毒散) combined with western medicine has made certain progress in the treatment of COVID-19, while PQR is more applicable to a wider range of people than ginseng. PQR is cold and tonic in nature, it is more suitable for patients with lung fever and physical weakness. As a kind of health care food, PQR is rich in protein, polysaccharide and other nutrients, and has anti-inflammatory, antiviral, immune enhancing and other pharmacological effects. Future scholars should explore the role of PQR in the prevention or treatment of COVID-19, or the possibility of using it as a post-COVID-19 dietary supplement.

### Other

PQR could also prevent premature ovarian failure by regulating prostaglandin biosynthesis and ovulation (Zhu et al., 2015), and promote penile erection by directly inducing dilation and relaxation of penile cavernous vessels (Murphy and Lee, 2002). In addition, PQR has the effects of anti-radiation (Liao et al., 2019), protecting liver and kidney (Jiang et al., 2020), strengthening the function of spleen and stomach (Gao et al., 1998; Long et al., 2019), anti-fatigue (Chang et al., 2018),and so on. However, previous scholars have seldom studied these pharmacological effects of PQR, and future scholars can further study its pharmacodynamic components, action targets and mechanism, so as to enrich the clinical practice of PQR.

PQR and its active ingredients not only have obvious effects on central nervous system and cardiovascular protection, but also have their own characteristics in other pharmacological effects. For example, ginsenoside Rg3 has anticancer activity, Re has antioxidant and antiemetic effects, and PF11, a unique saponin of PQR, has neuroprotective effect, caffeic acid and chlorogenic acid could help to protect the myocardial cells. Therefore, it is very important to study the biological activity of monomer, because it can exclude the existence of other components, and it is easy to clarify its exact pharmacological mechanism. However, there is a lack of in-depth study on the pharmacodynamic mechanism monomer compounds of PQR, and the mechanism of PQR in animals or human is not comprehensive enough. Moreover, there is a lack of investigation on the clinical optimal effective dose and safe dose. Other pharmacological effects such as immune regulation, anti-inflammatory, antihypertensive and so on are less studied, and the mechanism is not fully described. This provides a broad space for future scholars to study PQR. The purpose of this part is to summarize the modern pharmacological research of PQR, find out the shortcomings, and put forward the relevant solutions, so as to provide reference and direction for future scholars to study PQR.

## Toxicity

### Toxicity

1) There were two males and one female, aged 24, 28 and 45 y, respectively, with no history of disease, who chewed fresh PQR rhizomes planted at home with a chewing amount of about 0.5–1 g. After chewing about 5–10 min, they were sudden chest tightness, palpitation, abdominal distension, nausea and mild dizziness. Two cases were accompanied by obvious vomiting and cold sweat due to a large dosage. The results showed that the blood pressure was low and the heart rate was fast, and the electrocardiogram examination showed no abnormality. And there was no skin flush, rash, pruritus and other allergic reactions in all three patients. In this case, a little or even a small amount of fresh PQR root by oral administration cause poisoning, which is very rare (Ye and Wang, 2001). Therefore, people must be very careful not to taste the fresh PQR rhizome, so as not to induce similar poisoning. As for the toxic mechanism, it needs to be further explored and studied by futurologist.
2) Male, 3 g of PQR was taken 4 h after birth, and on the second day he developed symptoms of food refusal and convulsion. Physical examination: the face and the whole skin were obviously flushed, the muscle tension of limbs was slightly increased, and the skin was irritated with twitching, which was relieved automatically after 3–4 s. CT diagnosis: extensive cerebral edema and subarachnoid hemorrhage, consistent with the changes of brain CT caused by PQR poisoning. There are many causes of intracranial hemorrhage in newborns, and intracranial hemorrhage caused by PQR poisoning is rare (Zhao et al., 1999). However, it can be cured through blood transfusion, vitamin K supplementation and hemostatic agent.

### Sensitization

1) Female, 6-year-old, with a history of food allergy, atopic dermatitis and long-term asthma. A few minutes after inhalation of powdered PQR, she was referred to the emergency department for anaphylaxis (urticaria and respiratory symptoms). The skin punctum test (SPT) showed that she was allergic to PQR, and the basophil activation test showed that she was allergic to PQR. After treatment with salbutamol, dexamethasone and diphenhydramine, the symptoms were relieved quickly (Stephanie et al., 2018).
2) Male, 3-year-old, with a history of asthma, atopic dermatitis and conjunctivitis allergy. The symptoms of conjunctivitis appeared after contact with atomized powder PQR. After leaving the allergen, symptoms disappeared within 24 h. The SPT showed that he was allergic to PQR, but no IgE mediated allergic reaction was observed after oral administration of PQR (Stephanie et al., 2018).

The above studies indicated that the toxicity and sensitization cases of PQR were rare. “New Compilation of Materia Medica” recorded that PQR, sweet and spicy, cool nature, non-toxic. In addition to the above studies, the rest of the literature on the toxicity and safety of PQR and its preparations showed that PQR belongs to non-toxic substances with no acute toxicity, subacute toxicity or genetic toxicity, and can be used as a toxicological safe functional food (Shi et al., 2014). However, PQR unlike traditional Chinese medicine, has only been used in China for more than 200 years. The toxicological study of PQR has not been carried out in depth, and its safety remains to be studied. In the future, a large amount of clinical and animal data will be needed to verify its safety and make it better used as a medicinal and food homologous product in the clinical and health care field.

### Industrial Applications

In recent years, with the improvement of people’s living standard and the enhancement of health care awareness, it is a project beneficial to human health to develop active natural products with medical health care functions into industrial products with market value. PQR could eliminate fatigue, enhance memory, anti-arrhythmia, anti-myocardial ischemia, improve immunity, promote blood circulation, protect liver and kidney and other health functions. In recent decades, a number of patents have reported the potential development value of PQR. (As shown in Table 7).

**TABLE 7:** The Industrial applications of panacis quinquefolii radix.

Type	Patent name	Patent number	Health efficacy
Health-care medicine	A PQR oral liquid and its preparation technology	CN202010352639.1	It has the characteristics of nourishing and health care
	A preparation method of PQR red tea chewable tablets	CN201911343486.8	It is convenient to eat and quench thirst
	A preparation method of PQR tablets for tonifying kidney and relieving physical fatigue	CN201811220840.3	It has the function of tonifying kidney and relieving physical fatigue
	A kind of PQR weight reducing tea and its preparation method	CN201711147984.6	It has the effect of promoting food digestion, invigorating spleen and stomach, regulating intestines, reducing fat and reducing weight. It can lose weight and improve beauty
	An immunity enhancing capsule of PQR and herba epimedii	CN201811220548.1	It has the ability to relieve body fatigue and enhance immunity
	A traditional Chinese medicine composition for treating prostate and its preparation method	CN201710558946.3	It has comprehensive efficacy in the treatment of prostate
Health-care food	A preparation method of PQR tea-wine	CN202010034780.7	It has the function of reducing stress, protecting liver and kidney, cosmetology and strengthening body
	A preparation method PQR wine	CN201811330413.0	It has the effect of relieving thirst and resisting fatigue
	A formulation of slow release PQR red tea	CN111134212A	It has the characteristics of quench thirst, health care and improving immunity
	A soothe the nerves health drink and its preparation method	CN201911192027.4	It has the effect of calming the mind and helping sleep
	A preparation method of PQR red jujube compound health drink	CN201610608114.3	It has immune-boosting properties
	A preparation method of PQR red tea cake	CN201911388219.2	It has the characteristics of nourishing, health care, improving immunity and resisting fatigue
	A kind of PQR biscuit for relieving physical fatigue and its preparation method	CN201810936612.X	It has the advantage of restoring physical strength, relieving mental fatigue, improving the endurance and working efficiency under the exhausted state
	A method for making chicken soup of PQR and red jujube	CN201710490267.7	It has the characteristics of nourishing, health care, improving immunity and resisting fatigue
Health-care cosmetics	A manufacturing method of health care PQR lyophilized powder	CN201911343486.8	It has the advantages of good taste, delaying aging, whitening and beautifying
	An application of PQR fermentation liquid as skin care or skin care additive	CN201811223867.8	It can stimulate collagen formation and reduce collagen degradation, and improve red blood silk, wrinkles, acne and pigment
	A preparation method of PQR mask	CN201811235701.8	It has the advantages of moisturizing, natural, green, mild and does not irritate the skin and is not sensitive

In the field of health care, the buccal tablets, oral liquid and capsule of PQR can tonify the kidney, strengthen the spleen and benefit the stomach, moisten the intestines and defecate, relieve fatigue and enhance immunity (Jin, 2002; Wang, 2005). On January 2, 2020, The National Health Commission and the State Administration for Market Regulation listed PQR as a medicinal and food homologous product. In the application of food field,PQR provides people with some optional health food types, such as tea, wine, beverage, biscuit, medicated diet, etc. (Ma et al., 2010). PQR contains some potential antioxidant capacity, which can reduce the oxidation substances in the body and improve the oxygen absorption ability of skin. PQR also shows its potential as a health cosmetics, such as PQR lyophilized powder and PQR mask. PQR has been proved to have the functions of stimulating collagen formation and reducing collagen degradation, delaying aging, whitening and freckle removing. (Liu, 2018) At present, the industrial application of PQR involves many aspects, and a variety of products have been developed. These products better retain the nutritional, medicinal and health value of PQR, so that people’s daily consumption of food and the natural health care function of PQR achieve organic integration, in line with the current people’s requirements for healthy diet. Moreover, PQR is applied to cosmetic industry, which has great social and economic benefits.

Health care products industry has also played an important role in China’s economic development. PQR has been transformed from a medicinal plant into a medicinal and food homologous product, expanding the consumer population. Moreover, the positive development of health food industry can also promote the development of health industry and traditional Chinese medicine in the new era. Consumers also hope that through the use of health food, they can enhance their health, improve their physical functions and even prevent diseases. In addition, PQR is recognized as a kind of nutritive and health medicine and food homology product. In the processing of PQR, the stems and leaves of PQR are often treated as garbage, which can not be used reasonably. According to the data, the stems and leaves of PQR contain a large number of saponins, polysaccharides, flavonoids, which can play a good role in immune regulation and antioxidant. In the process of processing PQR, if the stems, leaves and berries are fully utilized and squeezed into juice, and then concentrated into PQR juice, the waste can be turned into treasure, which can avoid the waste of resources and increase the economic income. It is hoped that this part can provide ideas and directions for future scholars to fully develop and utilize the resources of PQR.

## Summary and Prospect

On January 2, 2020, The National Health Commission and the State Administration for Market Regulation listed PQR as a medicinal and food homologous product. This study summarized the botany, chemical composition, quality control, pharmacological, toxicity and industrial development of PQR. At present, the components isolated from PQR include saponins, volatile oils, flavonoids, sterols, polyacetylene, phenolic acids and other chemical components as well as proteins, carbohydrates, vitamins, fatty acid and other essential nutrients for human body. Pharmacological studies have shown that PQR has pharmacological effects such as neuroprotection, cardiovascular protection, immune regulation, anti-tumor, anti-diabetes, anti-hypertension and so on. Studies on the safety of PQR showed that PQR was a non-toxic substance and could be used as a toxicologically safe functional food, but it also had rare toxicity and sensitization. This paper summarizes the industrial development of PQR and finds that PQR has relatively high economic and social benefits, but there are still some important gaps in the scientific literature:

First, PQR as a high-grade health care products, the price is expensive, although there are artificial cultivation products, but still in short supply. Currently, the medicinal part of PQR is dried root. According to the determination, the content of saponins and polysaccharides in the leaves of PQR is more than 10%, which is twice as much as that in the root of PQR. As the price of *Panax quinquefolium* remains high and its resources are in short supply, future scholars can focus their research on the stems and leaves of PQR with lower price, so as to fully develop and utilize them and prevent resource waste.

Second, a large number of compounds have been isolated from PQR, but the current research on these compounds may be only the tip of the iceberg. The research on the chemical composition of PQR mainly focuses on the saponins, amino acids and trace elements while the analysis and function of organic compounds such as protein and fatty acid are relatively less. Moreover, it was found that a large amount of crude protein and crude polysaccharide could not be effectively used with the discharge of waste residue after extraction. In the future, scholars can try to develop new separation and determination methods to comprehensively measure and analyze PQR, so as to prevent its effective components from not being fully utilized.

Third, at present, the determination of compounds in PQR is limited to saponins, volatile oil and amino acids, and the quality markers of PQR were not elucidated. The analytical methods have the disadvantages of complex sample preparation, toxic organic solvents, soil pollution, and not green enough. In the future, scholars can consider introducing integrated quality evaluation method and Internet analysis technology to find quality markers of PQR. Instead of being limited to the existing methods, they can try to adopt the UPCC and other environment-friendly methods to carry out the multi-component quality control of PQR.

Fourth, as a health medicine, the pharmacological activity of PQR has been verified by scholars at home and abroad. However, there is a lack of in-depth study on the mechanism of action of PQR monomeric compounds, and the mechanism of PQR in animals is not comprehensive enough. Future scholars should combine the biological activity research and clinical application research of PQR to explore the material basis of its therapeutic effect. At the same time, the clinical optimal effective dose and safe dose of PQR also need to be investigated.

Fifth, although PQR has been widely used as a medicinal and food homologous product, there is still a lack of in-depth study on its safety. Although most studies show that PQR is a non-toxic substance and can be used as a safe functional food. However, there are still some cases showing that PQR has certain toxicity and sensitization. In the future, a large number of clinical and animal experiments are needed to verify its safety, so that it can be better used as a medicinal and food homologous product in clinical and health care fields.

In general, PQR has good biological activity and has developed a variety of products in health care medicine, health food, health cosmetics and other industrial fields. It is a valuable edible and medicinal resource worthy of attention. PQR has changed from medicinal plant to medicine and food homologous product, which has expanded consumer groups. The positive development of health care industry can also promote the development of health industry and traditional Chinese medicine in the new era. Consumers also hope that they can enhance their health, improve their physical functions and even prevent diseases through the use of health food. Further development of health products of PQR will undoubtedly be the focus of future research. However, the available health-related data on PQR are insufficient, and its clinical health value needs to be further verified.

In this study, the botany, phytochemistry, quality control methods, pharmacological effects, toxicity, industrial development of PQR were reviewed, and its current situation was analyzed, and its future development direction was prospected, so as to provide valuable reference for future scholars to develop and utilize PQR.

## Author Contributions

HJ, SL conceived of and designed the review; AH, WM, SW, JZ, HY, SZ, and XW searched the literature and downloaded the documents and made classification; AH wrote the paper; and LY and AH checked the chemical structures and formula and contributed comments for version of the manuscript. All authors read and approved the final manuscript.

## Funding

This work was financially supported by the National Natural Science Foundation of China (Grant Nos. 81973604, 81803690, and 81703684), Special Funds from the Central Finance to Support the Development of Local Universities, the National Natural Science Foundation Matching Project (No. 2018PT02), the Innovative Talents Funding of Heilongjiang University of Chinese Medicine (NO. 2018RCD25), the Postdoctoral Initial Fund of Heilongjiang Province (UNPYSCT 2017219), the University Nursing Program for Young Scholars with Creative Talents in Heilongjiang Province (No. UNPYSCT-2017215), the National Natural Science Foundation Matching Project (No. 2017PT01), the Natural Science Foundation of Heilongjiang Province (No. H2015037), the Heilongjiang University of Chinese Medicine Doctoral Innovation Foundation (No. 2014bs05), the Application Technology Research and Development Projects of Harbin Technology Bureau (No. 2014RFQXJ149), the Heilongjiang Postdoctoral Scientific Research Developmental Fund (No. LBH-Q16210 and LBH-Q17161), the Heilongjiang University of Chinese Medicine Doctoral Innovation Foundation (NO: 2013bs04).

## Conflict of Interest

The authors declare that the research was conducted in the absence of any commercial or financial relationships that could be constructed as a potential conflict of interest.

## Abbreviations

**Abbreviations:** AA, ascorbic acid; APP, amyloid protein; APX, ascorbate peroxidase; Ara, arabinose; AST, aminotransferase; CAT, catalase; CK, creatine kinase; cTnT, cardiac troponin T; CUS, chronic unpredictable stress; DA, dopamine; 2,3- and 2,5-DHBA, 2,3- and 2,5-dihydroxybenzoic acid; DOPAC, 3,4-dihydroxyphenylacetic acid; ERK1/2, kinases 1/2; ES, esterase; FAT/CD36, fatty acid translocation enzyme/cluster differentiation 36; Gal, galactose; Glu, glucose; GLUT4, glucose transporter four; GSH-PX, glutathione peroxidase; HDL, high-density lipoprotein; 5-HIAA, 5-hydroxyindoleacetic acid; HVA, homovanillic acid; Keap 1, Kelch-like ECH-associated protein one; LDH, lactate dehydrogenase; LDL, low-density lipoprotein; MA, methamphetamine; MDA, malondialdehyde; MMP-2, metalloproteinase-2; 3-NP, 3-nitropropionic acid; NR, nitrate reductase; PDG, protopanaxdiol; PF11, pseudoginsenoside-F11; POD, peroxidase; PPO, polyphenol oxidase; PQR, Panacis Quinquefolii Radix; PTG, Protopanaxatriol; Rha, rhamnose; ROS, reactive oxygen species; SOD, superoxide dismutase; TG, triglyceride; TH, tyrosine hydroxylase; TRAIL, necrosis factor-related apoptosis-inducing ligand; Xyl, xylose.
